# A specific dispiropiperazine derivative that arrests cell cycle, induces apoptosis, necrosis and DNA damage

**DOI:** 10.1038/s41598-023-35927-6

**Published:** 2023-05-29

**Authors:** Victor P. Liu, Wai-Ming Li, Jack Lofroth, Mehreen Zeb, Brian O. Patrick, Tina M. Bott, Chow H. Lee

**Affiliations:** 1 Department of Chemistry and Biochemistry, Faculty of Science and Engineering, University of Northern British Columbia, Prince George, BC V2N 4Z9 Canada; 2 Department of Chemistry, University of British Columbia, Vancouver, BC V6T 1Z1 Canada; 3 Department of Physical Sciences, MacEwan University, 10700-104 Avenue, Edmonton, AB T5J 4S2 Canada

**Keywords:** Cancer, Cell biology, Chemical biology, Drug discovery, Chemistry

## Abstract

Dispiropiperazine compounds are a class of molecules known to confer biological activity, but those that have been studied as cell cycle regulators are few in number. Here, we report the characterization and synthesis of two dispiropiperazine derivatives: the previously synthesized spiro[2′,3]-bis(acenaphthene-1′-one)perhydrodipyrrolo-[1,2-a:1,2-d]-pyrazine (SPOPP-3, **1**), and its previously undescribed isomer, spiro[2′,5′]-bis(acenaphthene-1′-one)perhydrodipyrrolo-[1,2-a:1,2-d]-pyrazine (SPOPP-5, **2**). SPOPP-3 (**1**), but not SPOPP-5 (**2**), was shown to have anti-proliferative activity against a panel of 18 human cancer cell lines with IC_50_ values ranging from 0.63 to 13 µM. Flow cytometry analysis revealed that SPOPP-3 (**1**) was able to arrest cell cycle at the G2/M phase in SW480 human cancer cells. Western blot analysis further confirmed the cell cycle arrest is in the M phase. In addition, SPOPP-3 (**1**) was shown to induce apoptosis, necrosis, and DNA damage as well as disrupt mitotic spindle positioning in SW480 cells. These results warrant further investigation of SPOPP-3 (**1**) as a novel anti-cancer agent, particularly for its potential ability to sensitize cancer cells for radiation-induced cell death, enhance cancer immunotherapy, overcome apoptosis-related drug resistance and for possible use in synthetic lethality cancer treatments.

## Introduction

The use of chemicals and radiation to induce DNA damage is the most commonly used method for cancer therapy. Recently, there is an increased interest in manipulating the cell cycle to induce mitotic catastrophe as a novel anti-cancer therapeutic strategy^1–5^. Mitotic catastrophe is characterized by cells which would normally be arrested in G2/M phase due to damage in DNA or mitotic spindle but falsely proceed to mitosis due to defective cell cycle checkpoints^6^. The end result is senescence or cell death via either apoptosis, necrosis or autophagy^7^. This strategy relies on the use of DNA damaging agents or radiation in combination with cell cycle checkpoint inhibitors. Indeed, several G2/M phase checkpoint inhibitors^1–5^, including irinotecan, a currently used chemotherapeutic drug for metastatic colorectal cancer, have shown potential to sensitize tumor cells to ionizing radiation. As such, the discovery of new compounds which cause G2/M cell cycle arrest remains an important area of cancer research^8–10^.

There are currently few known biologically active dispiropiperazine derivatives. One of which is prospidium chloride. This compound, also known as prospidine, has cytostatic, anti-inflammatory and immuno-suppressive properties^11–13^. It has been classified as an anti-neoplastic compound based on its ability to inhibit T and B cell mitogenesis during lymphoblastic transformation^11^, and to lower tumor volumes of carcinogen-induced mammary tumors in rats^12^. Currently, prospidium chloride is used as an anti-rheumatic drug in refractory rheumatoid arthritis^14^.

Despite previous reports of biologically active dispiropiperazine compounds, other chemical derivatives have not been sufficiently explored. Here, we report for the first time the anti-proliferative activity of spiro[2′,3]-bis(acenaphthene-1′-one)perhydrodipyrrolo-[1,2-a:1,2-d]-pyrazine (SPOPP-3, **1**), a previously synthesized dispiropiperazine derivative. SPOPP-3 (**1**) has anti-proliferative activity against a panel of human cancer cell lines and is capable of arresting cell cycle at G2/M phase, and inducing apoptosis, necrosis and DNA damage as well as disrupting mitotic spindle positioning.

## Results

### Synthesis of SPOPP-3 (1) and SPOPP-5 (2)

**Figure 1:**
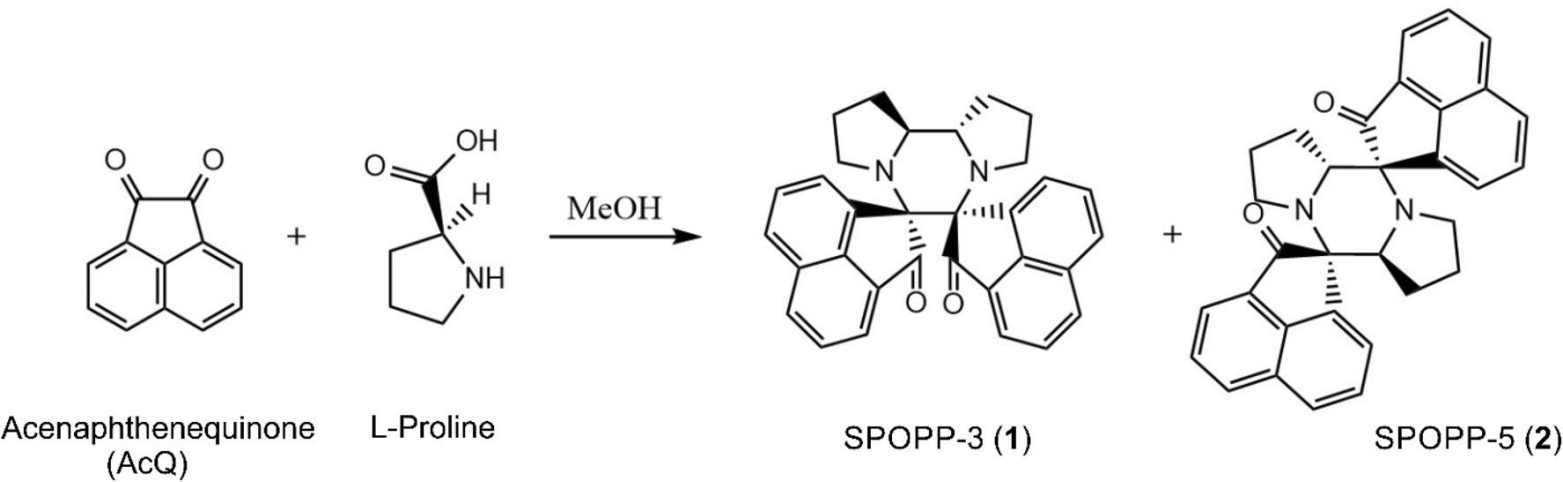
Synthesis of dispiropiperazine derivatives SPOPP-3 (**1**) and SPOPP-5 (**2**).

A recent report demonstrated the synthesis of two dispiropiperazine derivatives, spiro[2′,3]-bis(acenaphthene-1′-one)perhydrodipyrrolo-[1,2-a:1,2-d]-pyrazine (referred to here as SPOPP-3, **1**) and spiro[2′,5]-bis(acenaphthene-1′-one)perhydrodipyrrolo-[1,2-a:1,2-d]-pyrazine, through an azomethine ylide cycloaddition reaction using acenaphthenequinone (AcQ) and l-proline as substrates^15^. We performed a similar reaction with slight modifications as described in “Materials and methods” to obtain SPOPP-3 (**1**) (Fig. 1). Surprisingly, we also obtained a small quantity of spiro[2′,5′]-bis(acenaphthene-1′-one)perhydrodipyrrolo-[1,2-a:1,2-d]-pyrazine (referred to here as SPOPP-5, **2**) (Fig. 1 and Supplementary Figs. S2, S3, S6–S11), an isomer which has not been previously isolated due to its predicted unfavorable formation pathway^15,16^. Herein, we report for the first time, the purity (Supplementary Fig. S2) and structure of SPOPP-5 (**2**) as determined by FTIR (Supplementary Fig. S3), NMR (Supplementary Figs. S6–S10) and X-ray diffraction analyses (Supplementary Fig. S11).

### SPOPP-3 (1) reduced cell viability in human cancer cell lines

**Figure 2:**
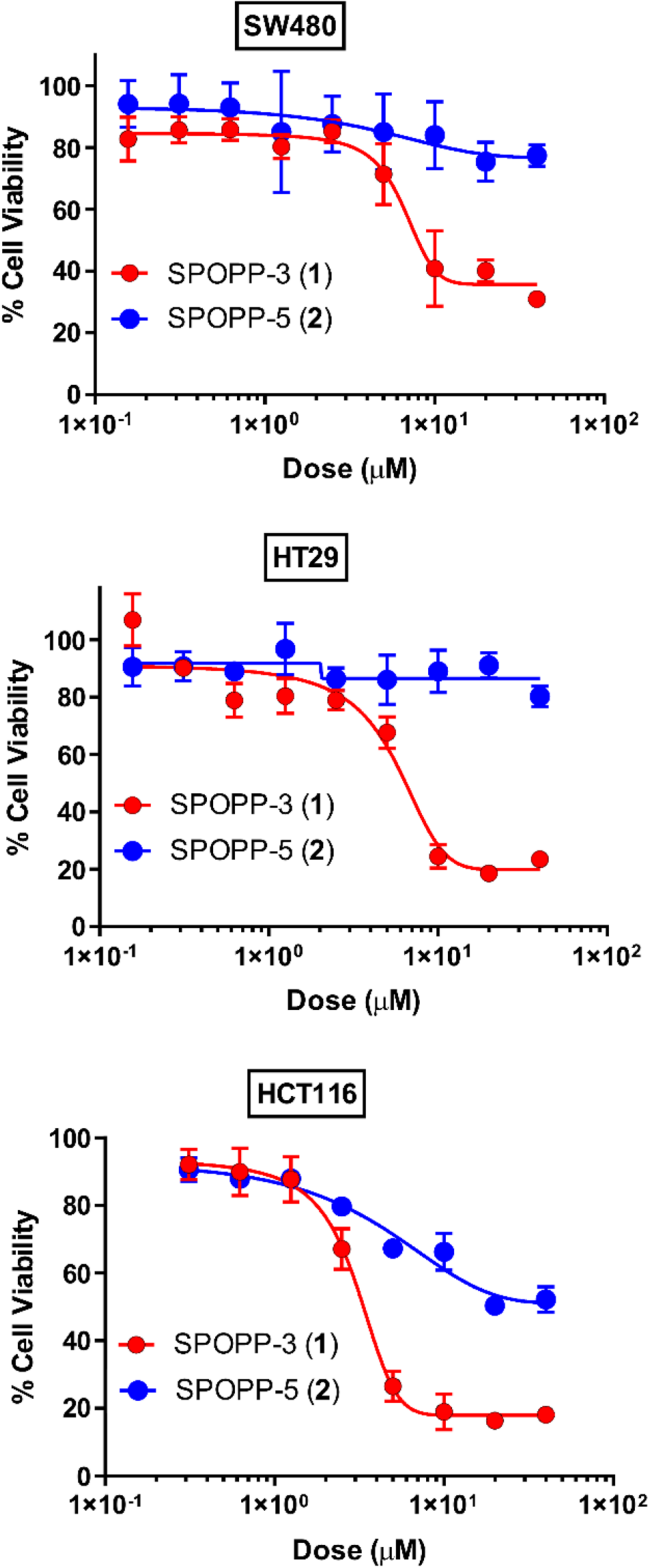
SPOPP-3 (**1**) inhibited human colon cancer cell viability. Cell viability in SW480, HT29 and HCT116 human colon cancer cells was assessed using the MTT assay. Cells were treated with different concentrations of SPOPP-3 (**1**) or SPOPP-5 (**2**) for 48 h. Results shown are representative from three separate experiments. Error bars are SEM.

To our knowledge, no bioactivity has been reported for SPOPP-3 (**1**). Herein, we show for the first time that SPOPP-3 (**1**) significantly reduced cell viability in human colon cancer cells (Fig. 2). For SPOPP-3 (**1**), the IC_50_ was 5.06 ± 1.43 µM, 5.42 ± 0.96 µM and 2.44 ± 0.83 µM in SW480, HT29 and HCT116 human colon cancer cells respectively (Table 1). In contrast, its isomer SPOPP-5 (**2**) had no significant effect, with IC_50_ > 100 µM (Fig. 2; Table 1). To determine whether SPOPP-3 (**1**) and SPOPP-5 (**2**) have different effects on different types of cancer cells, we assessed them on an additional 15 human cancer cell lines comprising another 8 different types of human cancers. Doxorubicin was used as a positive control on the cell lines and the summary is shown in Table 1. SPOPP-3 (**1**) remains inhibitory with IC_50_ values ranging from 0.63 ± 0.17 µM in human T lymphoblastoid cell line CEM to 13.0 ± 1.96 µM in human hepatoma cell line HepG2. Again, SPOPP-5 (**2**) showed insignificant activity in four additional cancer cell lines (MiaPaca-2, Panc-1, SKOV3 and MDA-MB-231) (Table 1). Based on the results that SPOPP-5 (**2**) had no significant anti-cell viability effect on 7 cancer cell lines, it was not further assessed on the other cell lines. The anti-proliferative effect of SPOPP-3 (**1**) was greatest against human leukemia cells lines (IC_50_ from 0.63 to 3.60 µM), human glioblastoma cell lines (IC_50_ from 2.95 to 6.30 µM), human colon cancer cell lines (IC_50_ from 2.44 to 5.42 µM), human cervical cancer cell lines (IC_50_ of 4.23 µM), human ovarian cancer cell lines (IC_50_ of 6.30 µM) and human breast cancer cell lines (IC_50_ from 4.00 to 6.17 µM). SPOPP-3 (**1**) has slightly weaker anti-proliferative activity against human liver cancer cell line (IC_50_ of 13.03 µM), human pancreatic cancer cell lines (IC_50_ from 8.62 to 9.17 µM) and human prostate cancer cell line (IC_50_ of 9.80 µM). Overall, the results showed that SPOPP-3 (**1**) has a relatively strong anti-proliferative effect on a panel of human cancer cell lines. To understand the mechanism whereby SPOPP-3 (**1**) exerts its anti-proliferative effect on cells, the following studies were conducted.

**Table 1:** IC_50_ of SPOPP-3 (**1**), SPOPP-5 (**2**) and doxorubicin against human cancer cell lines.

Cell lines	Types	IC_50_ (µM) of compounds		
		SPOPP-3 (1)	SPOPP-5 (2)	Doxorubicin
SW480	Human colon cancer	5.06 ± 1.43	> 100	0.22 ± 0.16
HT29	Human colon cancer	5.42 ± 0.96	> 100	0.26 ± 0.02
HCT116	Human colon cancer	2.44 ± 0.83	> 100	0.10 ± 0.05
MiaPaca2	Human pancreatic cancer	8.62 ± 3.18	> 100	0.25 ± 0.24
Panc1	Human pancreatic cancer	9.17 ± 2.67	> 100	0.43 ± 0.14
SKOV3	Human ovarian cancer	6.30 ± 1.35	> 100	0.04 ± 0.01
MDA-MB-231	Human breast cancer	6.17 ± 1.84	> 100	1.06 ± 0.03
MCF-7	Human breast cancer	5.76 ± 0.10	ND	0.03 ± 0.02
MCF-7-Adr	Human breast cancer	4.00 ± 0.85	ND	ND
T47D	Human breast cancer	4.76 ± 1.24	ND	ND
HepG2	Human liver cancer	13.03 ± 1.96	ND	0.36 ± 0.12
HeLa	Human cervical cancer	4.23 ± 1.29	ND	0.26 ± 0.12
DU145	Human prostate cancer	9.80 ± 1.94	ND	0.02 ± 0.01
K562	Human leukemia	3.60 ± 1.13	ND	ND
KG1a	Human leukemia	1.76 ± 0.84	ND	ND
CEM	Human leukemia	0.63 ± 0.17	ND	ND
U251	Human glioblastoma	2.95 ± 0.09	ND	0.01 ± 0.004
U87	Human glioblastoma	6.30 ± 1.72	ND	0.01 ± 0.002

The IC_50_ the data shown is an average taken from three independent experiments. *ND* not determined.

### SPOPP-3 (1) arrested cell cycle at G2/M phase

**Figure 3:**
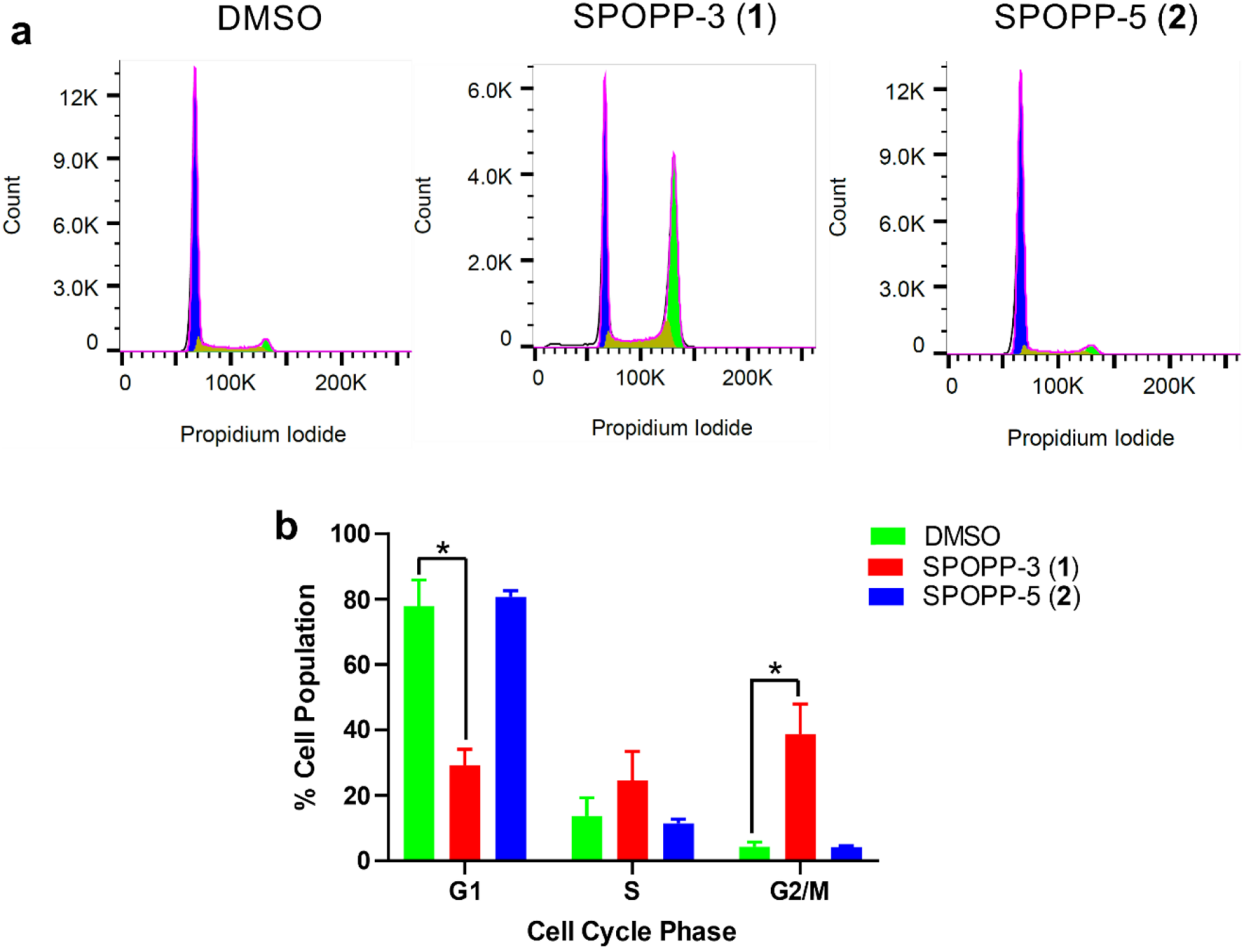
SPOPP-3 (**1**) arrested cell cycle at G2/M phase in SW480 cells. (**a**) SW480 cells were treated with 2% DMSO, 20 µM SPOPP-3 (**1**), or 20 µM SPOPP-5 (**2**) for 24 h after which cells were harvested and subjected to cell cycle analysis using flow cytometry. (**b**) The results from (**a**) and two another additional biological replicates (n = 3) were combined and expressed as shown. The cell cycle percentages were calculated based on the Watson Pragmatic model. Two-way ANOVA was performed: *p < 0.0001.

**Figure 4:**
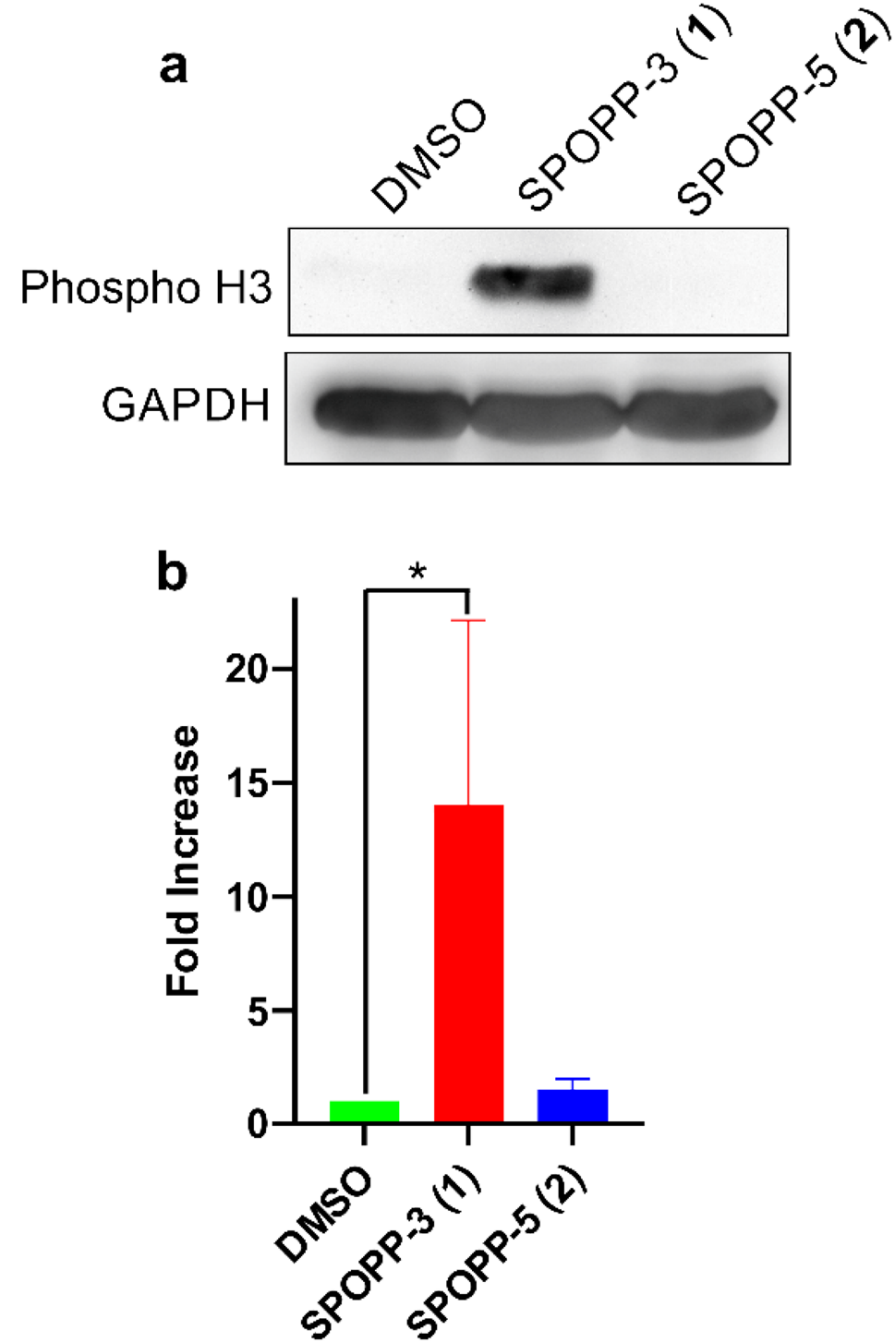
SPOPP-3 (**1**) induced phospho-histone H3 in SW480 cells. (**a**) Immunoblots showing phospho-histone H3 and GAPDH expression upon treatment with 2% DMSO, 20 µM SPOPP-3 (**1**) or 20 µM SPOPP-5 (**2**) for 24 h. (**b**) The results from (A) and another two biological replicate (n = 3) were averaged and expressed as shown. t-test was used: *p < 0.05.

**Figure 5:**
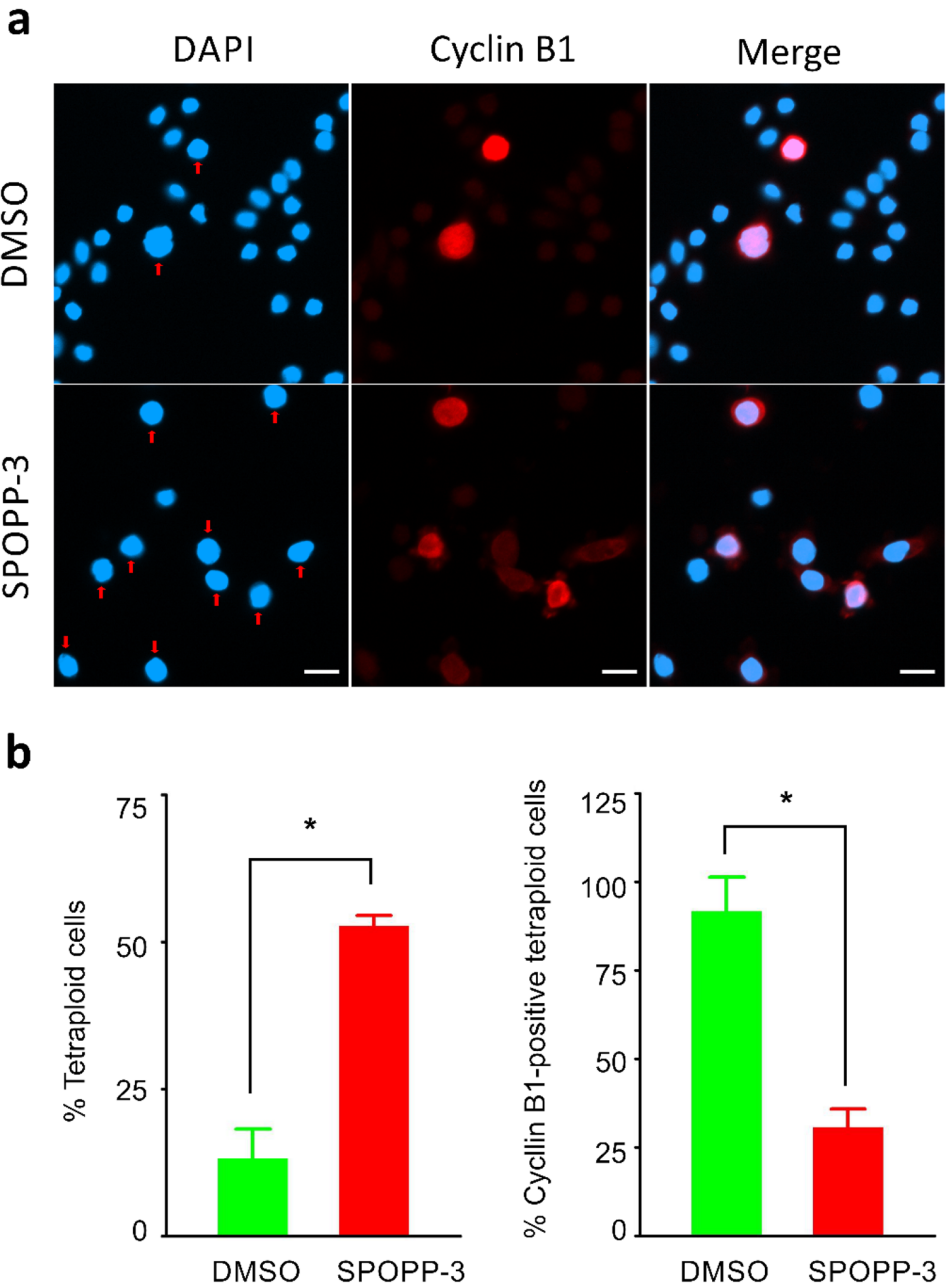
Defective cyclin B1 induction in SPOPP-3 treated cells as shown by immunofluorescence experiments. SW480 cells were treated with 40 µM SPOPP-3 or 2% DMSO for 24 h before fixation and immunostaining with cyclin B1 antibody and DAPI. (**a**) Representative image of cells treated with SPOPP-3 (lower images) showing a large population of tetraploid cells (indicated by arrows) without cyclin B1 staining. In contrast, DMSO-treated cells have a relatively small population of tetraploid cells all of which expressed cyclin B1. (**b**) Tetraploid cells (with doubled DAPI signal) (left) and the percentage of tetraploid cells positive for cyclin B1 (right) were quantified in each group and presented as average ± SD. One-way ANOVA was used: *p < 0.05. Scale bar 20 mm.

To determine the mechanism whereby SPOPP-3 (**1**) decreases cell viability, flow cytometry was performed for cell cycle analysis. As shown in Fig. 3, treatment with 20 µM SPOPP-3 (**1**) caused cell cycle arrest in the G2/M phase (Fig. 3b). No activity was observed for SPOPP-5 (**2**). To further investigate whether SPOPP-3 (**1**) induces arrest at G2 or M phase, we performed Western blot analysis to detect phospho-histone H3, an established sensitive mitotic marker^17^. Indeed, phospho-histone H3 level was clearly increased in SW480 cells treated with SPOPP-3 (**1**) but not SPOPP-5 (**2**) (Fig. 4), indicating that SPOPP-3 (**1**) arrests SW480 cells at the M phase of the cell cycle. To further investigate the effects of SPOPP-3 (**1**) on the cell cycle, we performed immunofluorescence experiments to study cyclin B1 activation. Cyclin B1 is one of the key factors in controlling entry into mitosis^18,19^ with its expression rapidly increased in G2 phase and peaking at late G2 or early M phase^20,21^. As shown in Fig. 5, the population of tetraploid cells in SPOPP-3 (**1**)-treated cells as indicated by cells having doubled DAPI signal, was significantly increased when compared with DMSO control. This confirms flow cytometry results that SPOPP-3 (**1**) caused cell cycle arrest in the G2/M phase where the cells failed to divide into daughter cells. In control cells, most of the tetraploid cells had significantly higher cyclin B1 staining compared to SPOPP-3-treated tetraploid cells (Fig. 5b). Thus, our results indicate that SPOPP-3 (**1**) treatment is associated with defective cyclin B1 activation.

### SPOPP-3 (1), but not SPOPP-5 (2), induced cell apoptosis and necrosis

**Figure 6:**
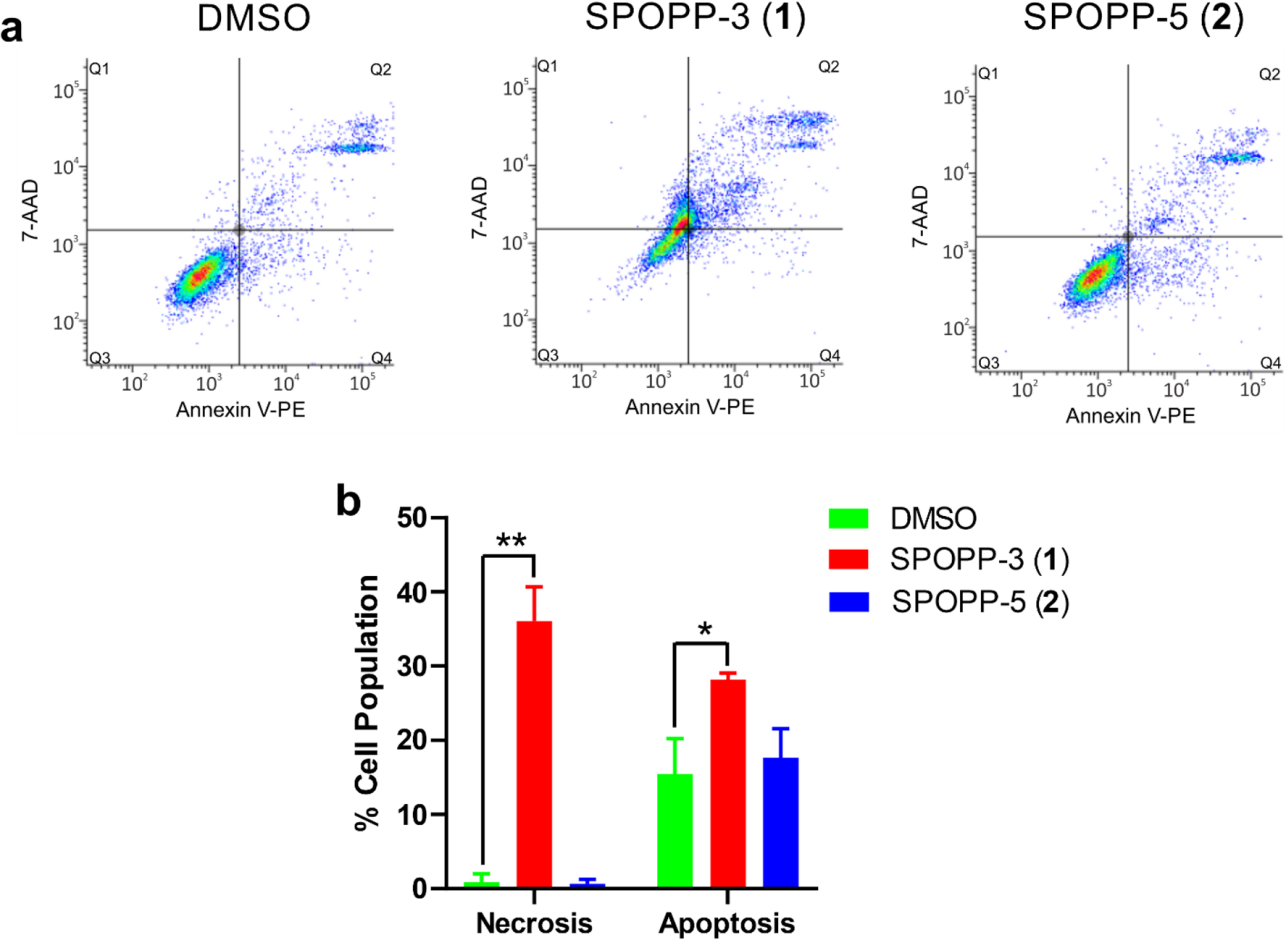
SPOPP-3 (**1**) induced apoptosis and necrosis in SW480 cells. (**a**) SW480 cells were treated with 2% DMSO, 20 µM SPOPP-3 (**1**), or 20 µM SPOPP-5 (**2**) for 24 h, after which cell lysates were isolated and subjected to cell death analysis using flow cytometry. (**b**) The results from (**a**) and two other additional biological replicates (n = 3) were combined and expressed as shown. Two-way ANOVA was used: *p < 0.005, **p < 0.0001.

To determine the possible effect of SPOPP-3 (**1**) on cell death that led to the observed decrease in cell viability, we used flow cytometry to analyse apoptosis and necrosis. The commonly used stains to detect necrosis and apoptosis are 7-AAD and Annexin V-PE, respectively. After treatment with SPOPP-3 (**1**), SPOPP-5 (**2**) or 2% DMSO for 24 h, cells were double stained with 7-AAD and Annexin V-PE. As shown in Fig. 6a, cells treated with SPOPP-3 (**1**) changed to a more necrotic state (Q1; 32.64%) as compared to the DMSO-treated cells (Q1; 0.5%), and this is statistically significant (Fig. 6b). SPOPP-3 (**1**) also significantly induced apoptosis (Q2 + Q4) in SW480 cells (Fig. 6). On the other hand, SPOPP-5 (**2**) had no significant effect on apoptosis or necrosis as compared to the control.

### Effect of SPOPP-3 (1) on mitotic spindle formation

**Figure 7:**
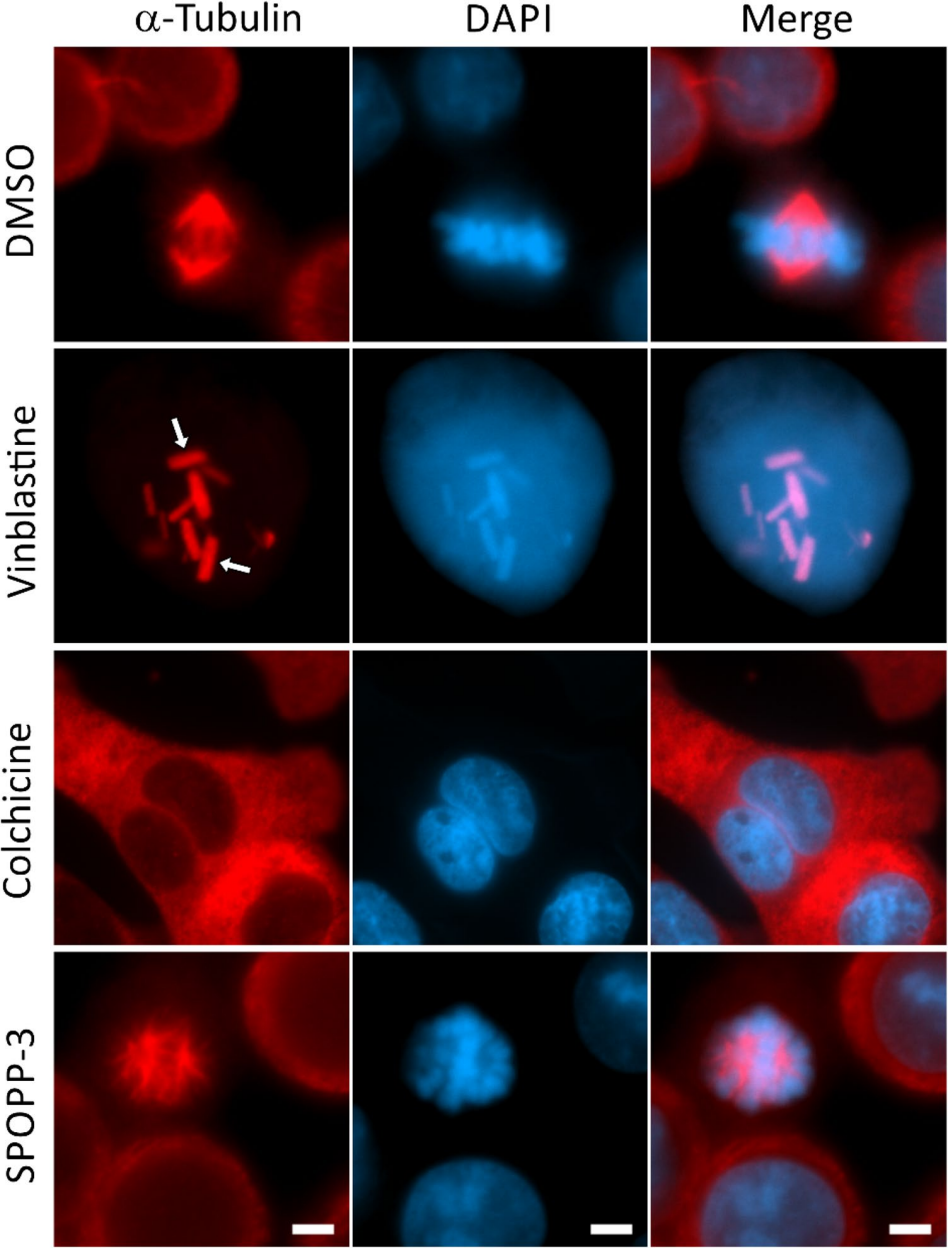
SPOPP-3 (**1**) does not cause microtubule disruption. SW480 cells were treated with 50 nM vinblastine, 1 µM colchicine or 40 µM SPOPP-3 (**1**) for 24 h before fixation and immunostaining with α-tubulin antibody and DAPI. Mitotic spindles were clearly observed in SPOPP-3 (**1**) treated cells while colchicine and vinblastine induced microtubule disruption via different mechanisms. Diffuse α-tubulin staining in the cytoplasm and para-crystal formation (arrows) can be observed with colchicine and vinblastine treatment respectively. Scale bar 5 mm.

Immunofluorescence studies were carried out to investigate the potential function of SPOPP-3 (**1**) as a microtubule toxin. Two drugs, vinblastine and colchicine which are well known to disrupt microtubules, were used as positive controls. Vinblastine, which belongs to the family of vinca alkaloids, binds to tubulin at a specific site and inhibits mitotic spindle formation leading to cell cycle disruption^22,23^. When used at high concentrations, vinblastine is known to cause paracrystal formation due to tightly packed tubulin aggregates^24^. Indeed, when SW480 cells were treated with 50 nM vinblastine, paracrystals were observed (Fig. 7). Colchicine, on the other hand, disrupted microtubules and caused diffuse α-tubulin staining throughout the cells (Fig. 7). In contrast, mitotic spindles could be observed in cells treated with SPOPP-3 (**1**). However, the positions of the mitotic spindles appeared to be disrupted when compared with control cells treated with DMSO (Fig. 7). Displacement of the mitotic spindles was also associated with the lack of chromosome alignment at the equator of the cell (Fig. 7). In summary, these results suggest that although SPOPP-3 (**1**) does not disrupt microtubule formation, mitotic spindle positioning appears to be affected.

### SPOPP-3 (1) induced DNA damage

**Figure 8:**
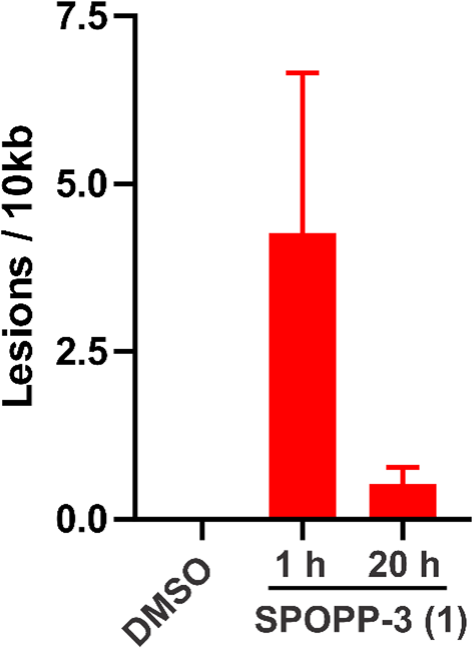
SPOPP-3 (**1**) induced DNA damage. DNA lesion quantification using LORD-Q in SW480 cells treated with 40 µM SPOPP-3 (**1**) for the indicated time periods. Short and long amplicons amplified from the mtDNA gene was used in the quantification of lesions in mitochondrial DNA. Data presented are averaged from three biological replicates ± SEM (n = 3).

Based on the findings that SPOPP-3 (**1**) causes G2/M arrest associated with cyclin B1 down regulation and mitotic spindle displacement, it may be possible that the activity of SPOPP-3 (**1**) is via DNA damage^19,25^. To investigate this possibility, we used the qPCR-based method (LORD-Q) to detect DNA lesions^26^. Since this method can detect DNA damage regardless of the type of DNA lesion, we reason that this is the most appropriate method. As shown in Fig. 8, although the data did not reach statistical significance because of the large variation in data, the effect of SPOPP-3 (**1**) on DNA damage can be detected as early as 1 h after treatment. However, such effect was much reduced after 20 h of treatment (Fig. 8).

## Discussion

In this study, we report the synthesis of SPOPP-3 (**1**) and show for the first time that it has strong anti-proliferative activity against 18 human cancer cell lines (Table 1). To our surprise, using the previously reported synthesis procedure^15,16^, we also obtained the structural isomer SPOPP-5 (**2**), a novel compound. We believe that SPOPP-5 (2) was formed in our hands because we used 35 °C and 3 h for the synthesis whereas Haddad et al.^15^ refluxed their synthesis reaction at 65 °C for 2 h. Lower synthesis temperature during cycloaddition reactions is known to favor more kinetically controlled product^16^. In contrast to SPOPP-3 (**1**), SPOPP-5 (**2**) had virtually no anti-proliferative activity (Table 1). We speculate that the configuration of the two carbonyl groups in SPOPP-3 (**1**), specifically in the absence of additional chemical components in between them, may contribute to its anti-proliferative activity (Fig. 1). Future structure–activity relationship studies would need to be conducted to test this hypothesis. The differential effect of SPOPP-3 on the different cancer cell lines could be related to the growth rate of the cell lines. For example, SPOPP-3 has stronger anti-proliferative effect on the faster growing human leukemia cell lines (K562, KG1a, CEM) and glioblastoma cell lines (U251, U87) but has weaker effect on the slower growing HepG2 liver cancer cell line, DU145 prostate cancer cell line, and pancreatic cancer cell lines (MiaPaca2, Panc1) (Table 1). Interestingly, this is not true for the positive control doxorubicin which showed strong anti-proliferative effect on both faster growing cells (U251, U87) and slower growing cells (DU145, SKOV3). Such observations suggested that SPOPP-3 and doxorubicin exerted their anti-proliferative effect through different mechanisms.

The anti-proliferative activity of SPOPP-3 (**1**) was associated with its ability to induce apoptosis and necrosis (Fig. 6), as well its ability to cause cell cycle arrest in the G2/M phase (Fig. 3). Using phosphorylated histone H3 as an M phase marker, it was shown that at least some cells were arrested in M phase (Fig. 4). Our results also showed that cyclin B1 expression was drastically reduced upon SPOPP-3 (**1**) treatment (Fig. 5). Although a rise in cyclin B1 expression in late G2 phase and its translocation to the nucleus is important for the initiation of mitosis, its depletion using siRNA knockdown does not cause cells to be arrested only in G2 phase as this can be explained by the redundant function of cyclin B2^18,27^. We also demonstrated using microscopy that while microtubules seem to be unaffected by SPOPP-3 (**1**) treatment, defects in the M phase including mitotic spindle positioning and condensed chromosome alignment, were observed (Fig. 7). This may be due to decreased cyclin B1 levels as it is known that cyclin B1 is normally recruited to centrosomes and kinetochores^28,29^. We further investigated whether SPOPP-3 (**1**) causes DNA damage as it has been documented that cyclin B1 levels are reduced as a result of DNA damage^30,31^. Indeed, our results suggest that similar to bleomycin^26^, Indeed, our results show that SPOPP-3 (**1**) causes DNA damage at an early stage which was detectible at 1 h but not at 20 h after treatment (Fig. 8). This is similar to the effect of bleomycin^26^ and is likely the result of the quick DNA damage repair response ^32^. As a consequence, such an early DNA damage event could lead to cyclin B1 reduction, cell cycle arrest, apoptosis and necrosis which are cellular processes that occur much later.

The anti-proliferative properties of SPOPP-3 (**1**) has important implications in cancer therapy. Synthetic lethality is a novel approach in cancer treatment^4,5^. Since SPOPP-3 (**1**) is a potent inducer of G2/M arrest, it has the potential to be used in combination with G2/M checkpoint inhibitors to trigger mitotic catastrophe which is currently viewed as a favorable treatment strategy to enhance cell death either via apoptosis, necrosis or autophagy^1–5^. Particularly, in most of melanoma cases where G1/S transition mediated by the cyclin-CDK4 pathway is defective and has increased dependence on the G2/M checkpoint to induce cell cycle arrest when exposed to DNA damage, SPOPP-3 (**1**) may have an added advantage in combination with G2/M inhibitors^4^. Secondly, we found that SPOPP-3 (**1**) is an inducer of necrosis. Several lines of investigations have provided evidence to support the role of necrosis in enhancing cancer immunotherapy and as a possible strategy to overcome the resistance of cancer cells to apoptosis^33,34^. To this end, it will be important to assess whether SPOPP-3 (**1**) has such prowess in inducing pro-inflammatory processes by means of releasing damage-associated molecular patterns such as high mobility group 1 (HMGB1) protein, a marker for necrosis. Release of such factors into the extracellular matrix may lead to activation of CD8+ leukocytes and promote anti-tumor immunity^34^.

It is also important to decipher further the mechanism whereby SPOPP-3 (**1**) arrests cells at G2/M phase. For instance, piperazine derivatives have been shown to generate reactive oxygen species (ROS) within cells^35^. Therefore, it would be of interest to investigate whether SPOPP-3 (**1**) can generate ROS leading to oxidative DNA damage and subsequent arrest at G2/M phase. In terms of necrosis, one of the modes of cell death detected in cells treated with SPOPP-3 (**1**), it has become increasingly clear that necrosis may not simply be an uncontrolled cellular process, but rather a regulated pathway commonly known as necroptosis^36^. Necroptosis has also been reported to increase cancer metastasis in certain cell lines^37^. The duality of necroptosis being anti- and pro-tumorigenic still requires investigation to determine in what context is necroptosis beneficial. Since we have found respectable anti-proliferative activity of SPOPP-3 (**1**) against a large panel of cancer cell lines, it would be integral to further study the mode of cell death in different cell lines.

In conclusion, we describe the synthesis and biochemical characterization of a specific dispiropiperazine derivative called SPOPP-3 (**1**) with strong anti-proliferative activity against a wide panel of human cancer cell lines. SPOPP-3 (**1**) is able to induce DNA damage, apoptosis, necrosis, arrests cell cycle at G2/M phase and disrupts normal mitotic spindle positioning. This study has laid the foundation for the further development of SPOPP-3 (**1**) for possible use as a chemical tool to perturb and understand cellular processes, as well as a potential anti-cancer compound. For the latter, SPOPP-3 (**1**) should be explored in synthetic lethality approach and as a necrosis-inducing anti-cancer compound.

## Methods and materials

### Synthesis and purification of SPOPP-3 (1) and SPOPP-5 (2)

We adopted the previously described method for synthesizing spiro[2′,3]-bis(acenaphthene-1′-one)perhydrodipyrrolo-[1,2-a:1,2-d]-pyrazine (SPOPP-3, **1**)^15,16^. A mixture of acenaphthenequinone (1.822 g, 10 mmol) and l-proline (1.151 g, 10 mmol; Sigma-Aldrich) were dissolved in methanol (200 mL) and heated at 35 °C for 3 h. The reaction was monitored by thin layer chromatography (TLC) using ethyl acetate:hexane (1:2; v/v), and the spots were visualized using UV light (254 nm). Typically, an orange precipitate was formed during the first hour which changed to an orange-brown color after an additional hour and to a dark brown color after completion of the reaction. Solvent was removed under reduced pressure leaving a dark brown powder (1.722 g). Crude product, containing both SPOPP-3 (**1**) and SPOPP-5 (**2**), was mixed with 2 g of normal silica in 200 mL methanol. Solvent was removed under reduced pressure and the silica mixture was dry-loaded onto a column. Purification by flash chromatography, using normal-phase silica (50 g) and a 9:1 mixture of hexanes and ethyl acetate at a flow rate of 12 mL/min, was used to isolate a mixture of SPOPP-3 (**1**) and SPOPP-5 (**2**). Subsequent purification with three runs of flash chromatography employing a total of 686 mg of crude product were conducted. An orange-coloured band eluting at 530–830 mL corresponding to Rf = 0.14 with 9:1 (hexanes:ethyl acetate) was dried under reduced pressure to produce 280 mg of semi-purified SPOPP-3 (**1**). SPOPP-3 (**1**) was subsequently purified for biological testing by HPLC using a Phenomenex Luna 5u Phenyl-Hexyl 4.60 mm × 250 mm column. A gradient from 80 to 92% acetonitrile for 6 min at a flow rate of 2 mL/min was used as the eluent conditions. For the semi-purified SPOPP-3 (**1**), a total of 12.5 mg was injected into the HPLC and 5 mg (16%) of purified SPOPP-3 (**1**) (at retention of 4.08 min) was obtained. A yellow-coloured band eluting at 320–405 mL corresponding to Rf = 0.24 (9:1 hexanes:ethyl acetate) produced 120 mg of purified SPOPP-5 (**2**). SPOPP-5 (**2**) was purified using the same HPLC conditions as SPOPP-3 (**1**). One hundred twenty mg of semi-purified SPOPP-5 (**2**) was injected into HPLC and 21 mg (3%) of purified SPOPP-5 (**2**) (at retention of 6.02 min) was obtained. HPLC–MS and NMR analyses were used to confirm the identity of SPOPP-3 (**1**) and SPOPP-5 (**2**). 1D and 2D NMR spectra were recorded on an Agilent/Varian Inova 400 MHz NMR spectrometer with a 5 mm Kimble NMR tube (Rockwood, TN, USA) at the University of Alberta or on a Bruker 600 MHz NMR with a cryoprobe at the University of British Columbia. All HPLC analyses were performed on Agilent 1260 Infinity Systems with UV detector, and mass spectrometry were done using Agilent 6120 Single Quad MS.

### X-ray crystallographic analysis

Single orange irregular crystals of SPOPP-5 (**2**) (50 mg) were recrystallized from acetonitrile (10 mL) by slow evaporation. Crystals were obtained on day 7. A suitable crystal with dimensions 0.22 × 0.20 × 0.11 mm^3^ was selected and mounted on a Bruker APEX II area detector diffractometer. The crystal was kept at a steady *T* = 90(2) K during data collection. The structure was solved with the ShelXT^38^ solution program using dual methods and Olex2^39^ as the graphical interface. The model was refined with XL^38^ using full matrix least squares minimization on *F*^2^.

### Cell culture

All cell lines were obtained from American Type Culture Collection except HT29 (colon adenocarcinoma) which was obtained from Dr. Ranjana Bird at UNBC. All cells were maintained in Eagle’s Minimal Essential Medium (Lonza) except the following: MiaPaca-2 and Panc-1 were maintained in Dulbecco’s Modified Eagle Medium (Lonza), while K562, KG1a and CEM were maintained in RPMI 1640 medium (Lonza). All media were supplemented with 10% fetal bovine serum (Life Technologies Inc.) and antibiotics.

### Cell viability assay

The cytotoxic MTT assay was used to assess cell viability as previously described^40^. Briefly, cells were plated at a density of 1.5 × 10^3^ cells/well in 96-well plates. After 24 h, cells were treated with SPOPP-3 (**1**) or SPOPP-5 (**2**) for 48 h with concentration range from 0.16 to 100 µM. Cells were treated with the positive control doxorubicin from 0.003 to 0.8 µM. All absorbance data were expressed relative to the control, 0.1% DMSO, taken as 100% cell viability.

### Preparation of cell lysates, immunoblot analysis and antibodies

SW480 cells were seeded in 6-well plates at a density of 3.0 x 10^5^ cells per well and then treated on the next day with 20 µM SPOPP-3 (**1**), 20 µM SPOPP-5 (**2**), or 2% DMSO for 24 h. Cell lysates were prepared as previously described^41^. For immunoblot analysis, protein samples were resolved on a 10% SDS-PAGE and transferred onto a nitrocellulose membrane. The phospho H3 antibody (Ser10) (D2C8, 1:1,000, Cell Signaling) was used with an overnight incubation at 4 °C. Anti-GAPDH (G8795, clone GAPDH-71.1, 1:20,000, Sigma) was also used. Anti-mouse IgM-HRP (sc-2064, 1:4000, Santa Cruz Biotechnology), anti-mouse IgG-HRP (W402B, 1:4000, Promega) and anti-rabbit IgG-HRP (W401B, 1:4000, Promega) were used as secondary antibodies. All blots were visualized with the FluorChem Q system (ProteinSimple). Densitometry analysis was performed using the AlphaView Q software (ProteinSimple).

### Flow cytometry apoptosis and cell cycle analyses

Cells were plated at 2.5 × 10^5^ cells/well in 6-well plates and treated with compounds as described above. Cells were trypsinized followed by centrifugation and washed twice with phosphate-buffered saline. Live cells were stained with PE Annexin V and 7-AAD according to the manufacturer’s instructions for Apoptosis Detection Kit I (BD Pharmingen) and analysed by flow cytometry using a BD FACSMelody cell sorter (BD Biosciences) and BD FACSChorus software (V 1.0). For cell cycle analysis, the BD cycletest plus DNA reagent kit was used to stain for DNA and the data was analysed using the software FlowJo.

### Immunofluorescence

SW480 cells were plated in 4-well cover glass chambers at a density of 15 × 10^4^ cells per well with 0.5 mL EMEM. The cells were treated with the indicated drugs for 24 h before fixation using 100% methanol (− 20 °C) for 10 min. Subsequently, the cells were blocked using PBS with 2% BSA and 0.1% Triton-x-100 and then stained with α-tubulin antibody (1:100; Ab4074; Abcam) or cyclin B1 antibody (D5C10, 1:200, cell signaling). Anti-mouse-AF594 and anti-rabbit-AF 594 (1:200; Molecular Probes) were used as secondary antibodies respectively. All antibodies used were diluted in PBS with 0.5% BSA and 0.1% Triton-x-100. All blocking and antibody incubation steps were conducted for 1 h at room temperature. After each antibody incubation step, the cells were washed 3 times (10 min each) with wash buffer containing 0.1% Triton-x-100 in PBS. For cyclin B1 immunofluorescence, cells were fixed in 4% paraformaldehyde for 15 min at room temperature. To stain for DNA, cells were incubated with DAPI dihydrochloride (300 nM diluted in PBS) for 5 min after immunostaining with the secondary antibody. All fluorescence images were taken using an inverted Zeiss Axio Observer Z1 microscope with a motorized stage, Zeiss Axiocam 503 mono camera, Colibri 2 multicolor LED light source, Plan-Apochromat 20×/0.8 M27 or Plan-Apochromat 63×/1.4 Oil DIC M27 as objectives. For the quantification of tetraploid cells, DAPI signal in each cell was quantified in each image using the region of interest function in the Zen software. Tetraploid cells were distinguished with a DAPI signal double that of the remaining cells in the same image. Three images (each with at least 36 cells) from each group were quantified. The population of tetraploid cells that were cyclin B1 positive was then determined in each image and presented as an average for each group. Vinblastine sulfate and colchicine (both purchased from Sigma) were included as positive controls for microtubule toxins.

### DNA damage quantification

To assess whether SPOPP-3 treatment causes DNA damage, a long-run real-time PCR-based method (LORD-Q) was used with modifications^26^. SW480 cells (2.5 × 10^6^) were treated in 6-well plates with DMSO (control) or 40 μM SPOPP-3 for the indicated time periods before being harvested for total DNA isolation using the DNeasy Blood and Tissue kit (Qiagen). LORD-Q method was carried out using the established primer sets for both the long and short amplicons for the mtDNA gene^26^. PCR efficiencies were calculated using 37.5, 18.75, 9.375 and 4.688 ng DNA as template per PCR reaction. The rtPCR reaction (15 μL total volume) consisted of 0.05 × ResoLight dye, 1 × KAPA2G Fast Hot Start ReadyMix, 500 nM of forward and reverse primer, and the aforementioned quantities of isolated DNA as template. For amplification of short amplicons as reference, Quantabio PerfeCTa^®^ SYBR^®^ Green FastMix^®^ was used. Real time PCR analysis was carried out using a Bio-Rad CFX96 system and data analysis was performed using CFX Maestro software. The number of lesions per 10 kb was calculated based on the previously established equation^26,42^. The data presented is averaged from 3 biological replicates ± S.E.M.

### Statistical analysis

The cytotoxic MTT assays were performed in triplicate (3 wells/treatment) and the absorbance reading from the respective control was taken as 100% cell viability. For immunoblot analysis, the densitometry of phospho-histone H3 bands were normalized to their respective GAPDH bands and then expressed relative to the DMSO control (taken as 1.0). One-way or two-way ANOVA was performed as indicated. Data are expressed as mean ± SD and were analysed using GraphPad Prism version 8.0.2 (La Jolla, CA) or t-test. Holm-Sidak test was used for post-hoc analysis. A p value < 0.05 was considered statistically significant.

## Supplementary Information

Supplementary Information.

## Data Availability

The datasets used and/or analysed during the current study are available upon request from the corresponding author.
