# Supplementary material for: A specific dispiropiperazine derivative that arrests cell cycle, induces apoptosis, necrosis and DNA damage
Source: Sci Rep. 2023 May 29;13:8674. doi: 10.1038/s41598-023-35927-6 (PMC10227070; doi:10.1038/s41598-023-35927-6)
Supplement: Supplementary file 1 — Supplementary Information. [file 41598_2023_35927_MOESM1_ESM.pdf]

A specific dispiropiperazine derivative that arrests cell cycle, induces apoptosis, necrosis and DNA damage

Victor P. Liu[1], Wai-Ming Li[1], Jack Lofroth[1], Mehreen Zeb[1], Brian O. Patrick[2], Tina M. Bott[3], and Chow H. Lee*[1]

[1]Department of Chemistry and Biochemistry, Faculty of Science and Engineering, University of Northern British Columbia, 3333 University Way, Prince George, British Columbia V2N 4Z9, Canada.
[2]Department of Chemistry, University of British Columbia, Vancouver, British Columbia V6T 1Z1, Canada.
[3]Department of Physical Sciences, MacEwan University, Edmonton, Alberta T5J 4S2, Canada.

***Corresponding author:** Department of Chemistry and Biochemistry, University of Northern British Columbia, 3333 University Way, Prince George, BC V2N 4Z9, Canada. Tel.: 250-960-5413; Fax: 250-960-5170; E-mail address: chow.lee@unbc.ca (Chow H. Lee).

Supplementary Information

Table of Contents

1. Characterization of SPOPP-3 (**1**) and SPOPP-5 (**2**)……………………………………………2

2. Supplementary Figures from HPLC, MS, FTIR and NMR analyses
Figure S1: HPLC-chromatogram, UV- and MS-spectra of SPOPP-3 (**1**)…………………………3
Figure S2: HPLC-chromatogram, UV- and MS-spectrum of SPOPP-5 (**2**)………………………4
Figure S3: FTIR spectrum of SPOPP-3 (**1**) and SPOPP-5 (**2**)……………………………………..5
Figure S4: ^{1}H-NMR spectrum of SPOPP-3 (**1**)……………………………………………………6
Figure S5: ^{13}C-NMR spectrum of SPOPP-3 (**1**)…………………………………………………..7
Figure S6: ^{1}H-NMR spectrum of SPOPP-5 (**2**)……………………………………………………8
Figure S7: ^{13}C-NMR spectrum of SPOPP-5 (**2**)…………………………………………………..9
Figure S8: COSY spectrum of SPOPP-5 (**2**)………………………………………………………10
Figure S9: HMQC spectrum of SPOPP-5 (**2**)………………………………………………………11
Figure S10: HMBC spectrum of SPOPP-5 (**2**)……………………………………………………..12
Figure S11: X-ray ORTEP drawing of SPOPP-5 (**2**)………………………………………………..13

3. Raw data
Figure S12: Raw data for Fig. 4a……………………………………………………………………14

4. References…………………………………………………………………………………………14

1. **Characterization of SPOPP-3 (1) and SPOPP-5 (2).**

Spiro[2',3]-bis(acenaphthene-1'-one)perhydrodipyrrolo-[1,2-a:1,2-d]-pyrazine (SPOPP-3, **1**) and Spiro[2',5]-bis(acenaphthene-1'-one)perhydrodipyrrolo-[1,2-a:1,2-d]-pyrazine (SPOPP-5, **2**) were synthesized as described in the Materials and Methods section.

SPOPP-3 (1)

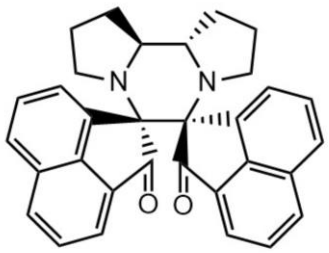

Orange powder; Rf = 0.33 (1 Ethyl acetate: 9 hexanes, ran twice); **LRESI-MS** $[M+H]^+$ m/z of 471 **^{1}H-NMR ($CDCl_3$, 600 MHz)** δ = 7.67 (app d, J = 8.1 Hz, 2H), 7.65 (app d, J = 7.0 Hz, 2H), 7.47 (app d, J = 7. 1 Hz, 2H), 7.43 (app t, J = 7.6 Hz, 2H), 7.39 (d, J = 8.2 Hz, 2H), 7.18 (app t, J = 7.7 Hz, 2H), 3.91-3.86 (m, 2H), 2.60 (td, J = 8.6, 2.9 Hz, 2H), 2.17-2.13 (m, 2H), 2.0 1-1.95 (m, 2H), 1.82- 1.75 ((m, 2H), 1.73-1.67 (m, 2H), 1.64-1.57 ppm (m, 4H); **^{13}C-NMR ($CDCl_3$, 125 MHz)** δ = 207.3, 141.4, 136.9, 134.1, 130.8, 130.2, 127.9, 127.7, 124.8, 123.3, 119.4, 72.3, 60.5, 47.6, 27.9, 21.3 ppm.
This is a known compound and our data is consistent with the published data of the compound.[1]

SPOPP-5 (2)

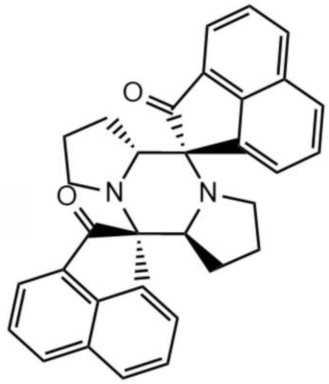

$C_{32}H_{26}N_2O_2$: Yellow powder, R = 0.53 (1 ethyl acetate: 9 hexanes, resolved twice on the same TLC); **LRESI-MS** $[M+H]^+$ m/z of 471. **^{1}H-NMR ($CDCl_3$, 400 MHz)** δ = 8.16-8.13 (m, 4H), 7.97-7.86 (m, 4H), 7.75 (m, 4H), 4.88 (dd, J = 8.4, 5.7 Hz, 2H), 2.58-2.36 (m, 4H), 1.62-1.30 (m, 6H), 0.66-0.49 ppm (m, 2H**). ^{13}C-NMR ($CDCl_3$, 100 MHz)** δ = 208.4, 141.5, 140.4, 131.8, 131.3, 129.9, 128.3, 127.6, 124.0, 121.2, 120.2, 74.6, 60.3, 45.7, 24.4, 24.4 ppm.

2. Supplementary Figures.

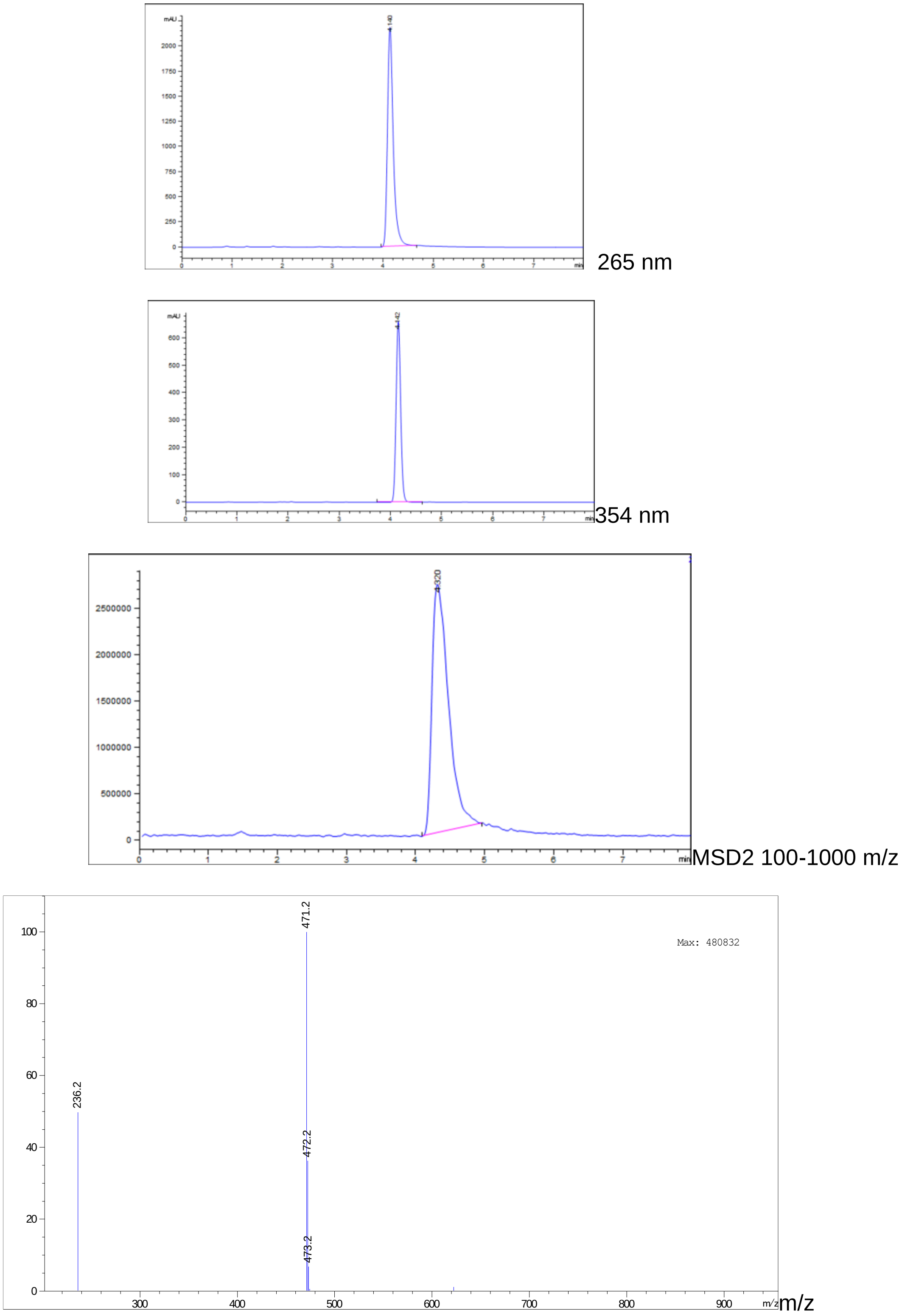

Figure S1: HPLC-chromatogram (265 and 354 nm), and MS-spectra of SPOPP-3 (**1**). The ESIMS spectrum exhibited a $[M + H]^+$ peak at *m/z* = 471.2.

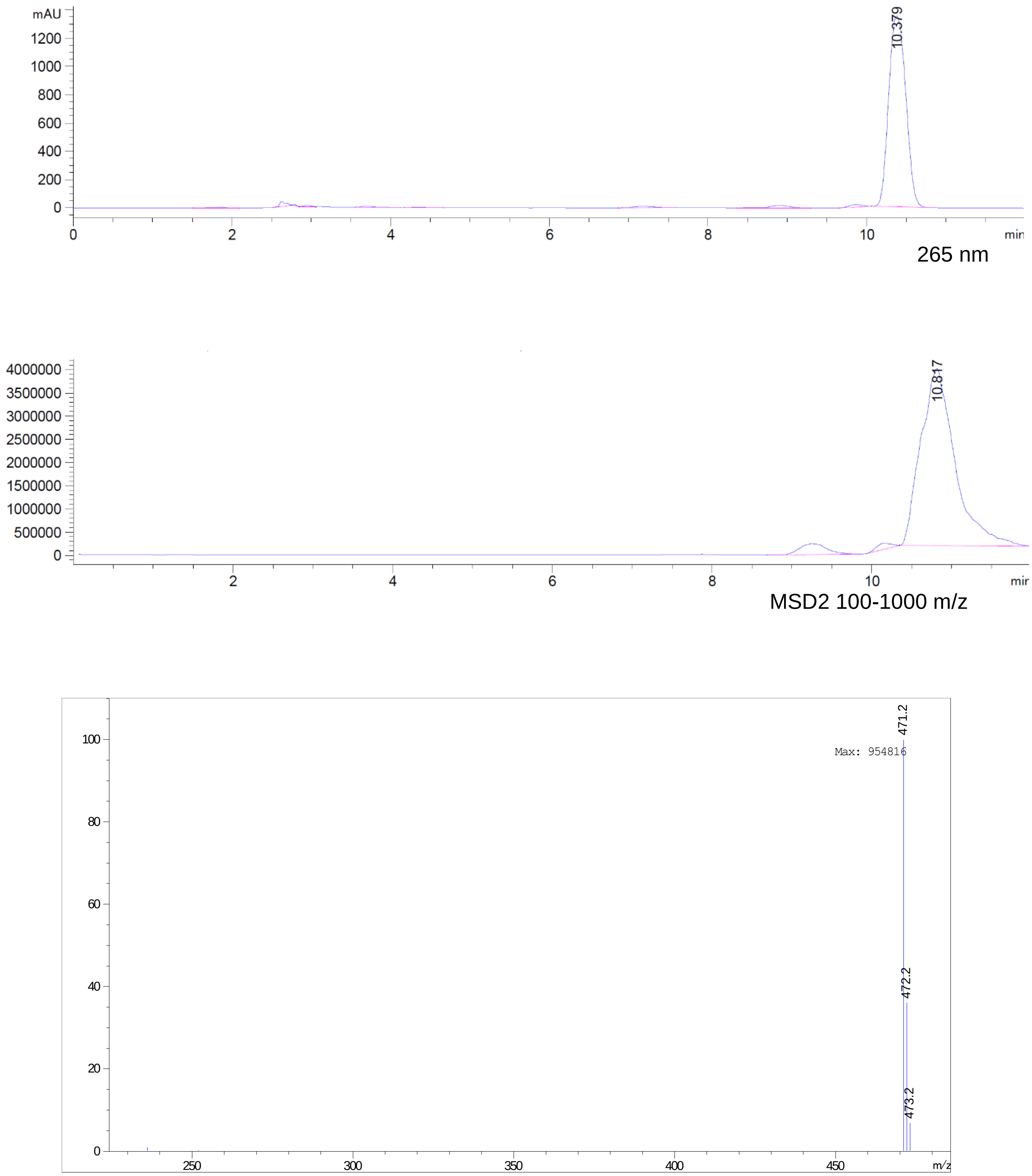

Figure S2: HPLC-chromatogram (265 nm), and MS-spectra of SPOPP-5 (**2**). The ESIMS spectrum exhibited a $[M + H]^+$ peak at *m/z* = 471.2.

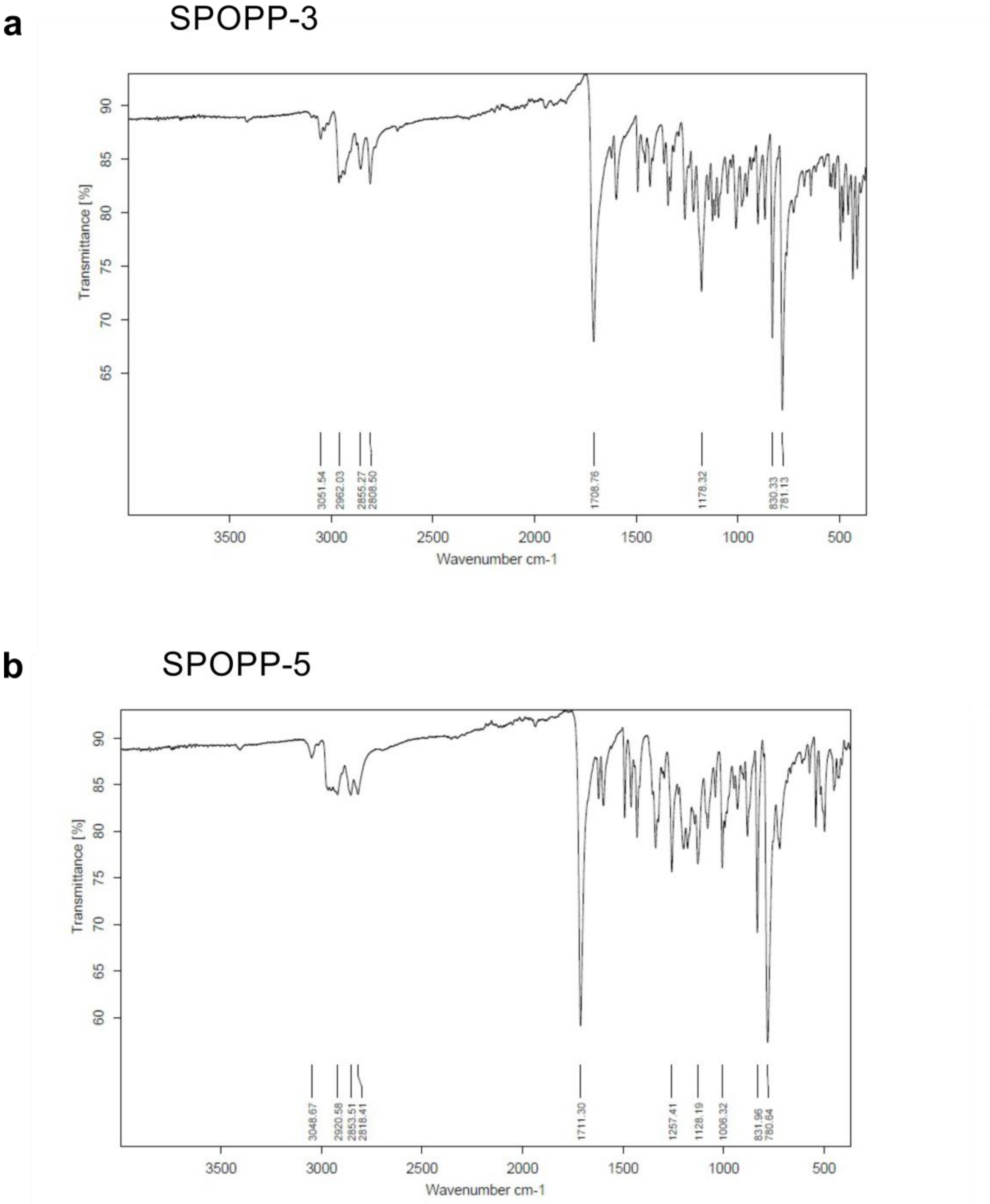

Figure S3: FTIR spectrum of (A) SPOPP-3 (**1**) and (B) SPOPP-5 (**2**). For SPOPP-3 (**1**), the C-H stretch can be seen between 2808.50 to 3051.54 cm^{-1}. The typical C=O absorption is also noted at 1708.76 cm^{-1}. Similarly, SPOPP-5 (**2**) has the notable C-H stretch from 2818.41 to 3048.67 cm^{-1} and the C=O absorption is at 1711.30 cm^{-1}. The difference in the two molecules are also evidenced by the absorption intensity differences in the fingerprint region. The peak at 1178.32 cm^{-1} for SPOPP-3 (**1**) is the notable differentiating absorption peak.

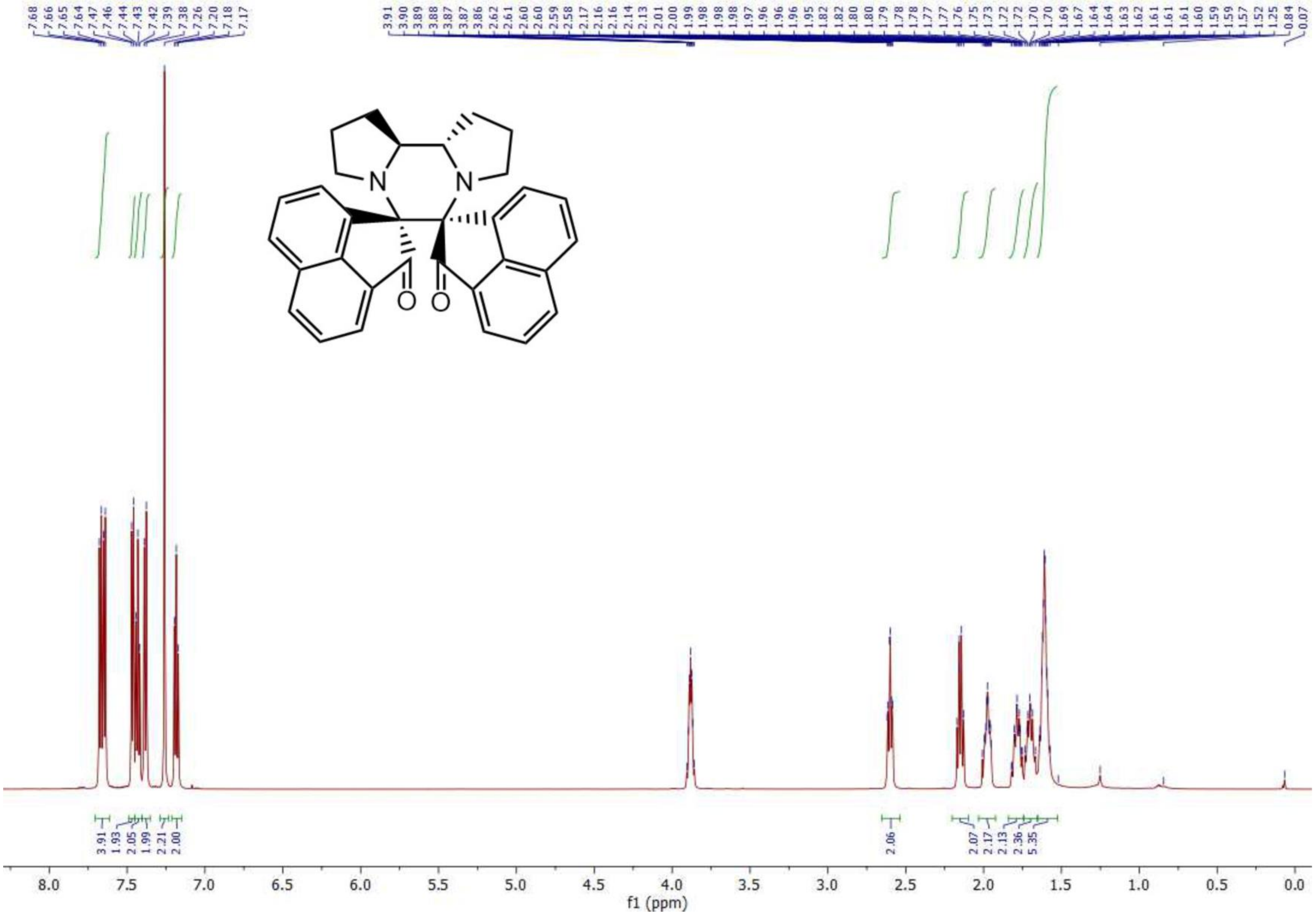

Figure S4: ^{1}H-NMR spectrum of SPOPP-3 (**1**) ($CDCl_3$ with 0.3% TMS, 600 MHz).

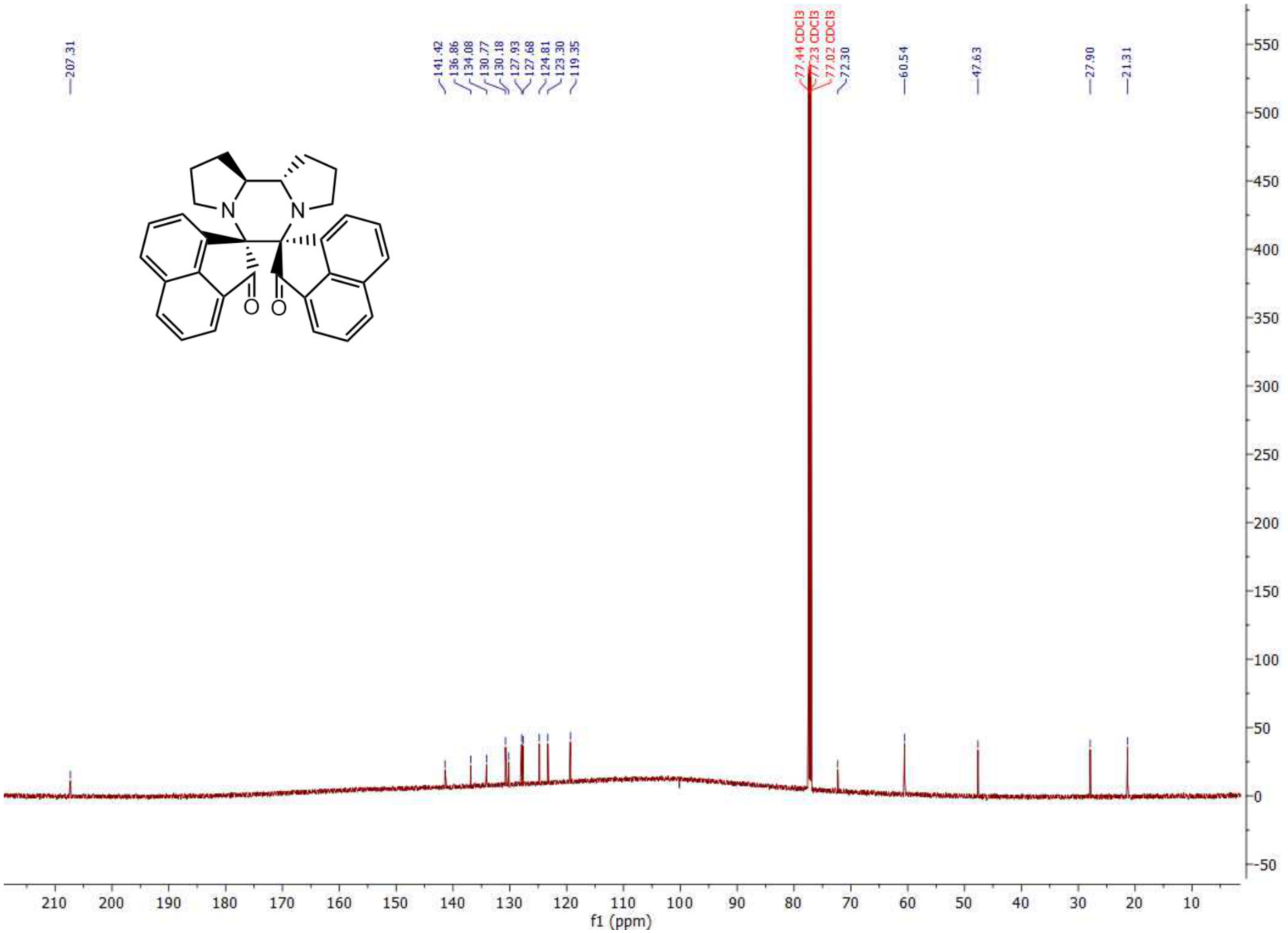

Figure S5: ^{13}C-NMR spectrum of SPOPP-3 (**1**) ($CDCl_3$ with 0.3% TMS, 600 MHz).

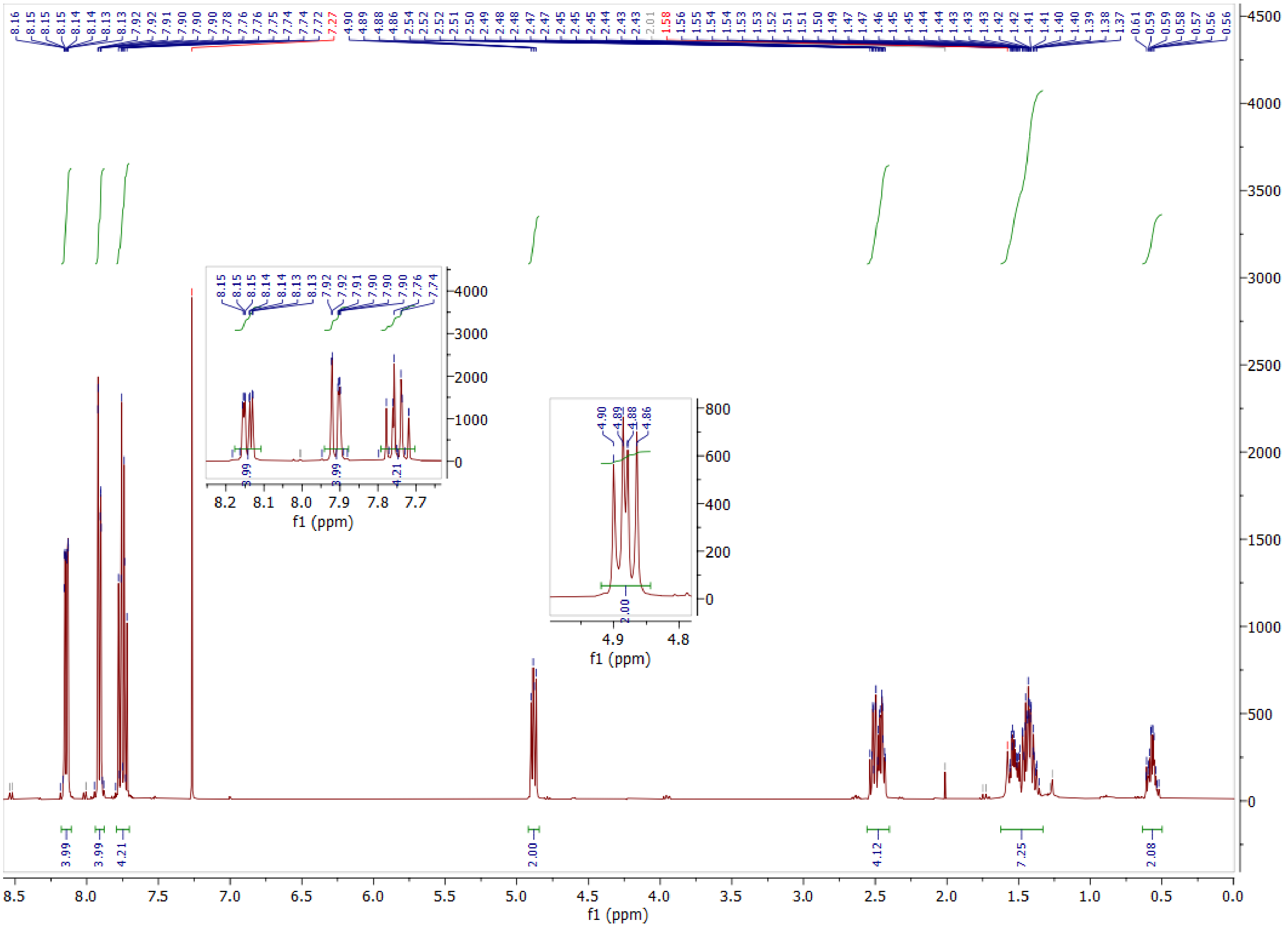

Figure S6: ^{1}H-NMR spectrum of SPOPP-5 (**2**) ($CDCl_3$ with 0.3% TMS, 400 MHz)

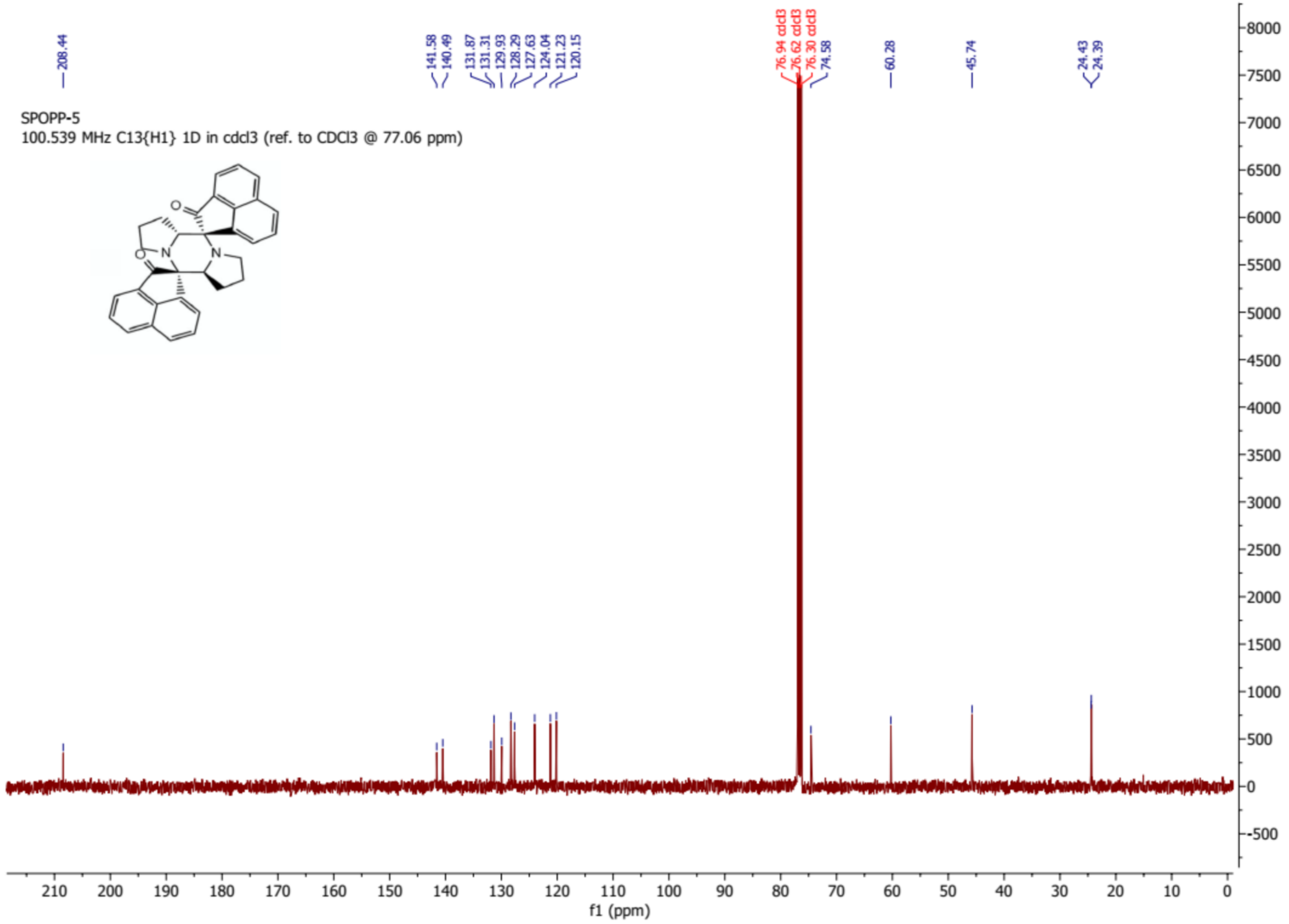

Figure S7: ^{13}C-NMR spectrum of SPOPP-5 (**2**) ($CDCl_3$ with 0.3% TMS, 100 MHz)

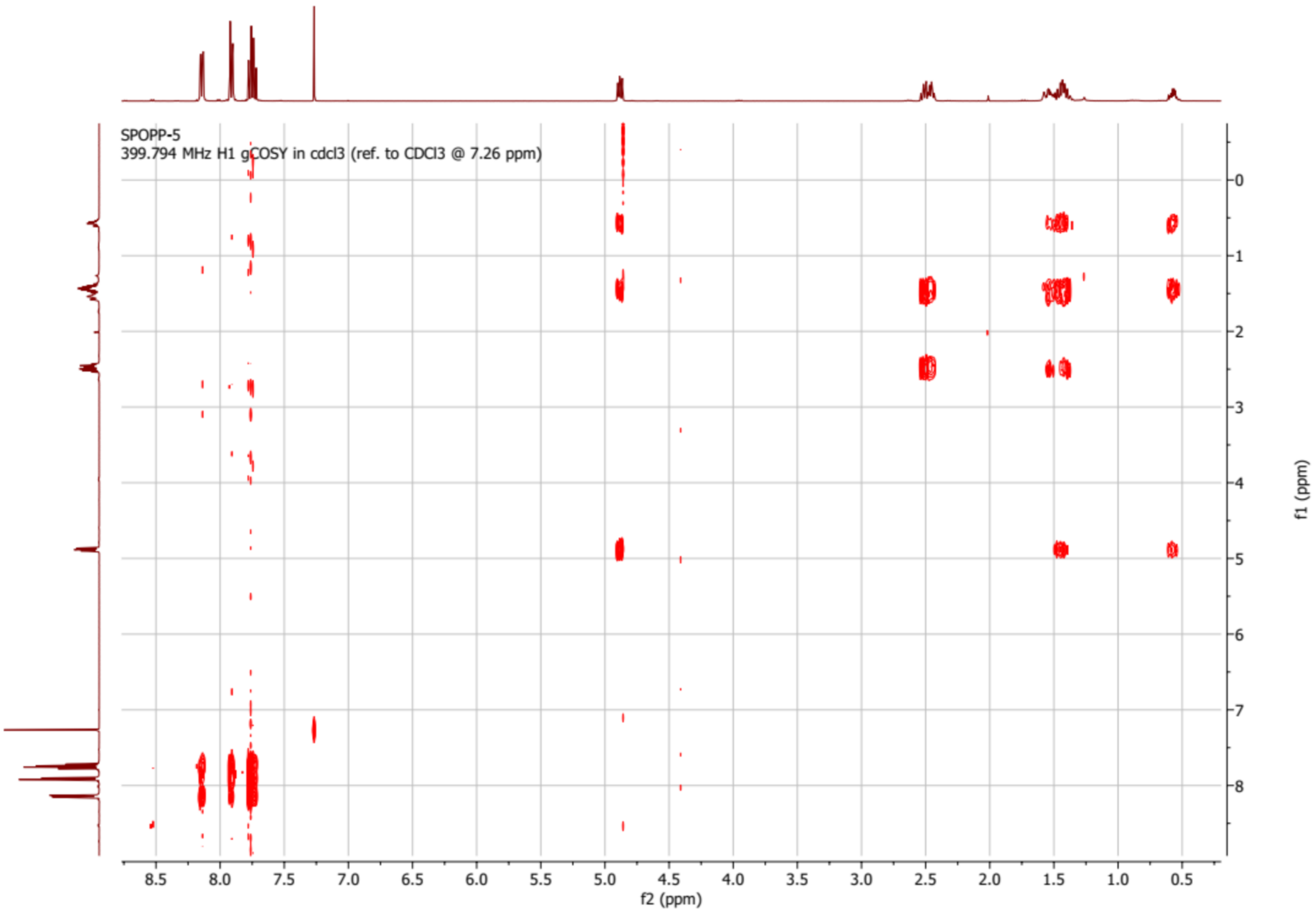

Figure S8: COSY spectrum of SPOPP-5 (**2**) ($CDCl_3$ with 0.3% TMS, 400 MHz).

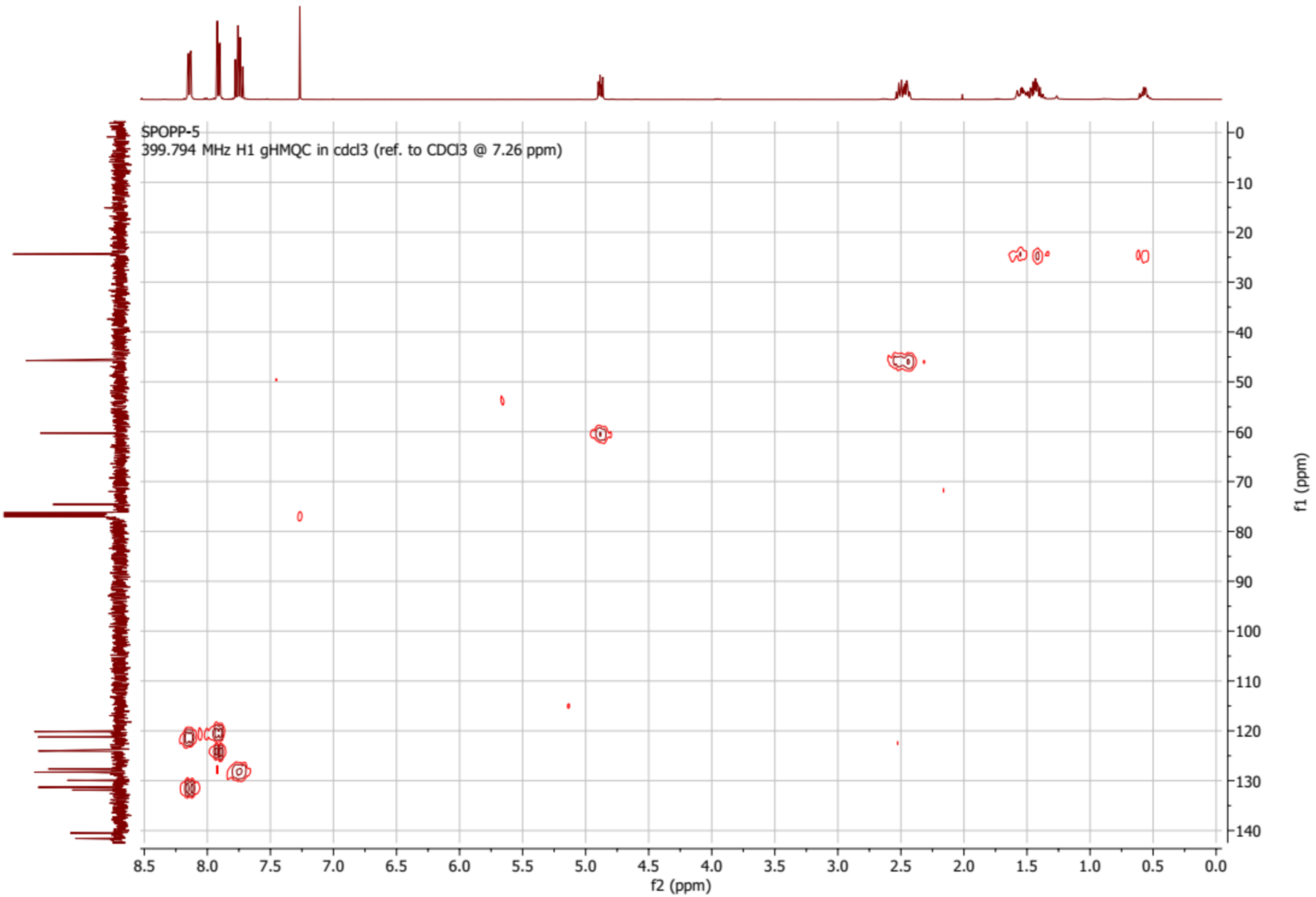

Figure S9: HMQC spectrum of SPOPP-5 (**2**) ($CDCl_3$ with 0.3% TMS, 400 MHz).

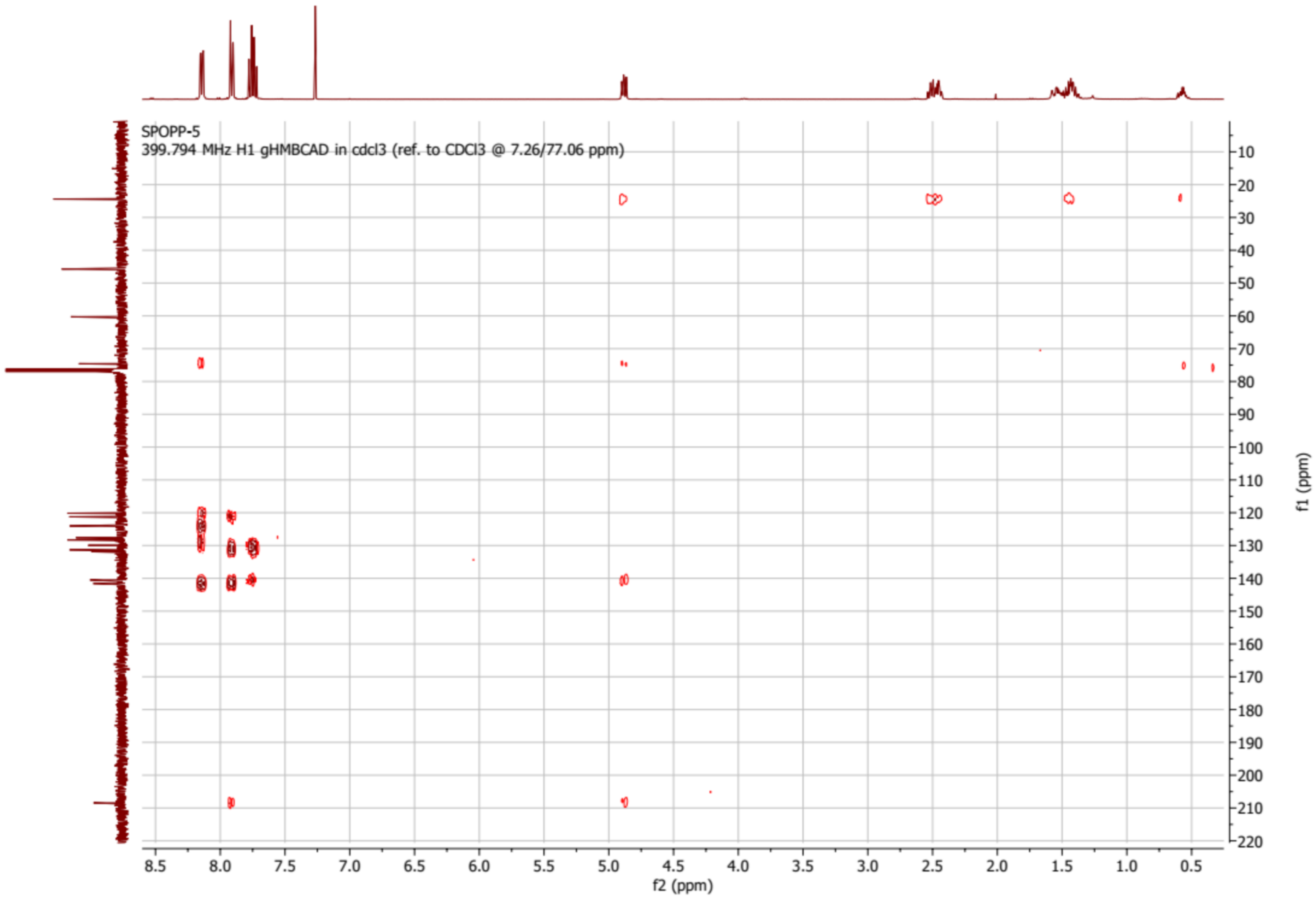

Figure S10: HMBC spectrum of SPOPP-5 (**2**) ($CDCl_3$ with 0.3% TMS, 400 MHz)**.**

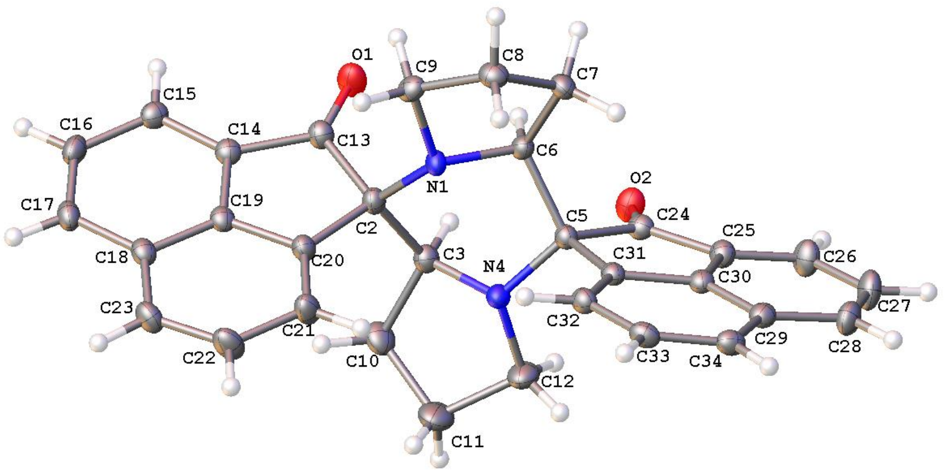

Figure S11: X-ray ORTEP drawing of SPOPP-5 (**2**).

Crystal Data. $C_{32}H_{26}N_2O_2$, M_r = 470.55, monoclinic, $P2_1/c$ (No. 14), a = 11.7680(4) Å, b = 13.4613(4) Å, c = 15.0138(5) Å, β = 97.045(2)°, $\alpha = \gamma$ = 90°, V = 2360.42(13) Å^3, T = 90(2) K, Z = 4, Z' = 1, μ(CuK$_\alpha$) = 0.652, 29690 reflections measured, 4166 unique (R_{int} = 0.0472) which were used in all calculations. The final wR_2 was 0.0957 (all data) and R_1 was 0.0367 (I > 2(I)).
X-ray crystallographic data of SPOPP-5 (**2**) (CIF)

**Compound**	**SPOPP-5**
Formula	$C_{32}H_{26}N_2O_2$
$D_{calc.}$/ g cm^{-3}	1.324
μ/mm^{-1}	0.652
Formula Weight	470.55
Colour	orange
Shape	irregular
Size/mm^3	0.22×0.20×0.11
T/K	90(2)
Crystal System	monoclinic
Space Group	$P2_1/c$
a/Å	11.7680(4)
b/Å	13.4613(4)
c/Å	15.0138(5)
α/°	90
β/°	97.045(2)
γ/°	90
V/Å^3	2360.42(13)
Z	4
Z'	1
Wavelength/Å	1.54178
Radiation type	CuK$_\alpha$
Θ_{min}/°	3.785
Θ_{max}/°	66.711
Measured Refl's.	29690
Ind't Refl's	4166
Refl's with I > 2(I)	3660
R_{int}	0.0472
Parameters	325
Restraints	0
Largest Peak	0.204
Deepest Hole	-0.208
GooF	1.036
wR_2 (all data)	0.0957
wR_2	0.0917
R_1 (all data)	0.0422
R_1	0.0367

3. Raw data.

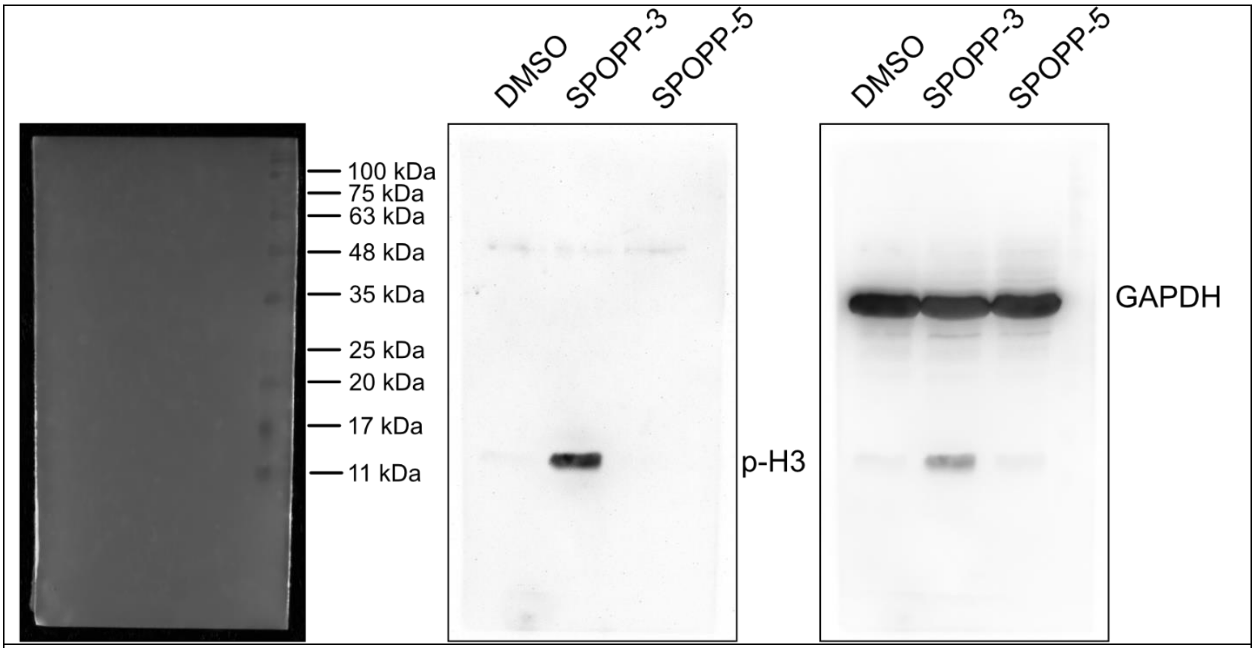

Raw Data for Fig 4a. Blot was probed with anti-phosho-histone H3 (middle image). Blot was then reprobed with anti-GAPDH (right image). The left image shows where the protein markers are.

Figure S12: Raw data for Fig. 4a.

4. References

1. S. Haddad, S. Boudriga, F. Porzio, A. Soldera, M. Askri, M. Knorr, Y. Rousselin, M. M. Kubicki, C. Golz and C. J. Strohmann, *J. Org. Chem.,* 2015, **80,** 9064-9075.
